# Role of *Thymus ciliatus* (Thyme) to Ameliorate the Acute Neurotoxicity Induced by Bisphenol A: *In Vivo* Supported with Virtual Study

**DOI:** 10.3390/ph18040509

**Published:** 2025-03-31

**Authors:** Dallal Kourat, Djallal Eddine H. Adli, Mostapha Brahmi, Faisal K. Alkholifi, Faten F. Bin Dayel, Wafaa Arabi, Marie-Laure Fauconnier, Bakhta Bouzouira, Khaled Kahloula, Miloud Slimani, Sherouk Hussein Sweilam

**Affiliations:** 1 Laboratory of Biotoxicology, Pharmacognosy and Biological Valorization of Plants (LBPVBP), Department of Biology, Faculty of Sciences, University of Dr Moulay Tahar, Saida 20000, Algeria; dallal.kourat@univ-saida.dz (D.K.); djillou2006@yahoo.fr (D.E.H.A.); wafaaarabi@yahoo.fr (W.A.); bombixc2@yahoo.fr (K.K.); mslimani20@gmail.com (M.S.); 2 Department of Biological Science, Faculty of Natural and Life Sciences, University of Relizane, Relizane 48000, Algeria; 3 Department of Pharmacology, College of Pharmacy, Prince Sattam Bin Abdulaziz University, Al-Kharj 11942, Saudi Arabia; f.alkholifi@psau.edu.sa (F.K.A.); f.bindayel@psau.edu.sa (F.F.B.D.); 4 Laboratory of Natural Molecules Chemistry, Gembloux Agro-Bio Tech, University of Liège, 2 Passage des Déportés, B-5030 Gembloux, Belgium; marie-laure.fauconnier@uliege.be; 5 Department of Pathological Anatomy and Cytology, CHU of Sidi Bel Abbes, Sidi Bel Abbes 22000, Algeria; bakhtabouzouira@gmail.com; 6 Department of Pharmacognosy, College of Pharmacy, Prince Sattam Bin Abdulaziz University, Al-Kharj 11942, Saudi Arabia; 7 Department of Pharmacognosy, Faculty of Pharmacy, Egyptian Russian University, Badr City 11829, Egypt

**Keywords:** *Thymus ciliatus*, GC-MS, bisphenol A, neurobehavioral tests, virtual screening

## Abstract

**Background/Objectives:** The purpose of this research was to investigate the effects of bisphenol A (BPA) exposure on neurobehavioral testing in young Wistar rats and to evaluate the therapeutic potential of *Thymus ciliatus* (TEO) essential oil to attenuate the damage induced by this chemical toxin. **Methods**: The essential oil was extracted by hydro-distillation (yield of 2.26%), and the characterization by GC-MS indicates that the major components of *Thymus ciliatus* oil are thymol (63.33%), p-cymene (13.4%), and σ-terpinene (6.69%). Acute BPA intoxication was induced with a dose of 50 mg/kg orally for 60 days. The neurobehavioral evaluation, performed using a comprehensive set of tests including the forced swim test, dark/light box, Morris water maze, open field test, and sucrose preference test, clearly demonstrated that bisphenol A (BPA) exposure induced significant neurobehavioral impairments. **Results**: These impairments included reduced exploratory behavior indicative of heightened stress, anxiety, and depressive-like states, as well as deficits in memory and learning. Furthermore, BPA intoxication was associated with metabolic disturbances such as hyperglycemia along with histopathological evidence of brain tissue damage. However, TEO treatment attenuated these adverse effects by restoring neurobehavioral function. Molecular docking analysis revealed an affinity between the major essential oils identified in *T. ciliatus*, BPA, and the 5HT2C receptor and the MAO, AChE, and BChE enzymes, suggesting a potential mechanism underlying BPA’s effects on behavior and memory. In addition, TEO also showed an interaction with these molecules, suggesting a therapeutic potential against BPA. These findings underscore the promising role of TEO in mitigating the poisonous effects of BPA and pave the way for additional research into the molecular mechanisms and therapeutic uses of natural bioactive compounds for the prevention and treatment of toxic diseases. Thymol, the major compound in TEO, exhibited activity related to the dopamine and serotonin pathways, so it could have potential antidepressant properties. **Conclusions**: Thymol might be a promising candidate for the treatment of neurodegenerative and neurological disorders such as depression, Parkinson’s disease, and Alzheimer’s disease while also preventing histological damage in the brain.

## 1. Introduction

Numerous studies in human populations have identified environmental pollutants as potential risk factors in the development of various diseases [1,2,3]. Exposure to low concentrations of endocrine-disrupting chemicals (EDCs) has been shown to interfere with multiple metabolic processes, leading to significant damage to bodily tissues [1,4,5].

Recent research suggests that EDCs, including halogenated aromatic hydrocarbons, can disrupt hormonal regulation and affect other essential physiological functions [6]. A well-known example of an EDC is bisphenol A (BPA), an industrial chemical widely used in the production of various consumer goods such as food cans, water bottles, dental sealants, food storage containers, and thermal papers [7,8]. BPA has been shown to interact with multiple hormone receptors, exhibiting the ability to mimic, block, modify, or otherwise influence hormonal activities regulated by the brain [9]. Growing evidence indicates that BPA exposure can impair reproductive health, development, metabolism, immune responses, and neurobehavioral functions [10]. Furthermore, studies have demonstrated that even low doses of BPA can induce oxidative stress in key organs, including the brain, liver, kidneys, and reproductive system [10].

Essential oils (EOs) derived from plants have found widespread applications in aromatherapy, pharmacy, perfumery, cosmetics, and food preservation. This is attributed to their diverse and potent biological activities [11,12]. Essential oils are complex, volatile, naturally occurring substances with low molecular weights and distinct aromas [13,14]. The chemical classifications of essential oils from aromatic and therapeutic plants include alcohols, aldehydes, ketones, ethers/oxides, esters, amides, amines, phenols, heterocyclic compounds, and, predominantly, terpenes [13,15]. Essential oils rich in terpene alcohols exhibit the second-highest antibacterial activity, followed by those containing phenols or aldehydes, such as cinnamaldehyde, citral, carvacrol, eugenol, and thymol [13,16]. *Thymus ciliatus* (thyme) has long been esteemed for its culinary and medicinal qualities. In addition to its antimicrobial effects, this herb has exhibited anti-inflammatory, antioxidant, and anti-carcinogenic properties [17,18].

Based on the available bibliographic data, our research aims to estimate the incidence of bisphenol A (BPA) intoxication and its effects on the behavior of young Wistar rats, focusing particularly on their learning and memory performance. Additionally, we will evaluate the impact of treatment with *Thymus ciliatus* essential oil (TEO) through a neurobehavioral approach. This in vivo study is further supported by in silico docking analysis.

## 2. Results

### 2.1. Yield and Principal Components of Essential Oil (TEO)

**Figure 1:**
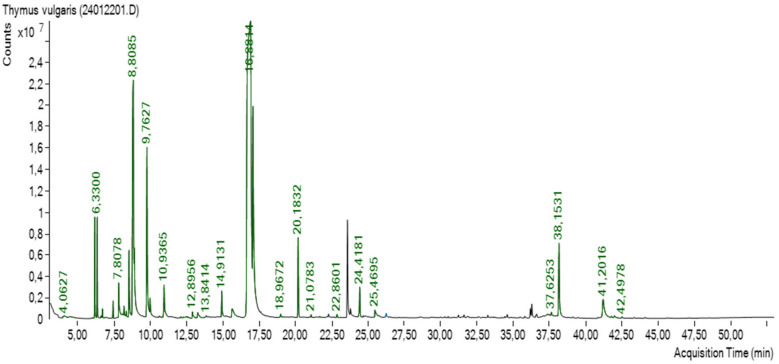
GC-MS chromatographic profile of TEO.

*Thymus ciliatus* essential oil (2.26% yield) was analyzed by gas chromatography, which allowed the identification of 17 components representing 98.55% of the total. The major components were thymol (63.33%), *p*-cymene (13.4%), and *σ*-terpinene (6.69%), respectively (Table 1 and Figure 1).

### 2.2. Neurobehavioral Tests

#### 2.2.1. Dark/Light Test

**Figure 2:**
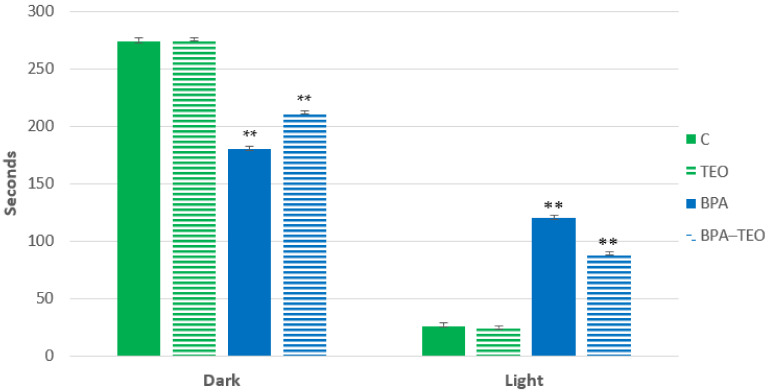
Comparison of TEO’s effects on anxiety in BPA-exposed rats. Data are presented as mean ± SEM. ** *p* < 0.01 (C vs. BPA, BPA vs. BPA–TEO); *n* = 7 per group. The results are expressed as the mean and standard error of the mean with ANOVA, followed by the Student–Newman–Keuls post hoc test, where significance is defined as ** *p* < 0.01 with analyses performed using Sigma Stat software v4.0.

The results revealed that rats intoxicated with BPA (BPA group) spent significantly more time in the lighted compartment compared with the control group (C) (*p* < 0.01), indicating anxiety behavior. Conversely, a significant increase (*p* < 0.01) in the duration spent in the dark compartment was observed in BPA-intoxicated rats treated with TEO (BPA–TEO group) compared with the BPA group, suggesting that thyme essential oil may help mitigate anxiety-related behavior (Figure 2).

#### 2.2.2. Y-Maze Test

**Figure 3:**
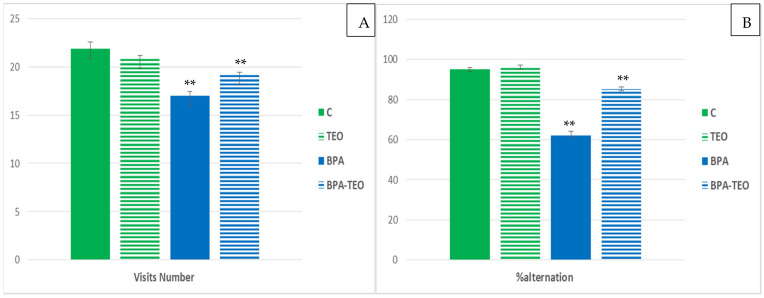
Number of visits (**A**) and alternation percentage (**B**) in control rats (C), control rats treated with TEO (TEO), BPA-intoxicated rats (BPA), and BPA-intoxicated rats treated with TEO (BPA–TEO). Data are presented as mean ± SEM. ** *p* < 0.01 (C vs. BPA); ** *p* < 0.01 (BPA vs. BPA–TEO). *n* = 7 per group. The results are expressed as the mean and standard error of the mean, with ANOVA, followed by the Student–Newman–Keuls post hoc test, where significance is defined as ** *p* < 0.01 with analyses performed using Sigma Stat software.

This test is a common method for evaluating working memory in laboratory animals. Statistical analyses of the Y-maze test results demonstrated that the mnemonic performance of rats exposed to bisphenol A (BPA) differed significantly from that of the control rats. Specifically, the alternation percentage (Figure 3A) and the number of visits (Figure 3B) made by the BPA-exposed rats during the test were significantly lower compared with the control rats (*p* < 0.01). Furthermore, the administration of TEO to the BPA-exposed rats was found to significantly improve their mnemonic performance, as evidenced by the increased percentage of alternations and the number of visits, which serves as an indicator of general activity, during the test (*p* < 0.01).

#### 2.2.3. Forced Swimming Test

**Figure 4:**
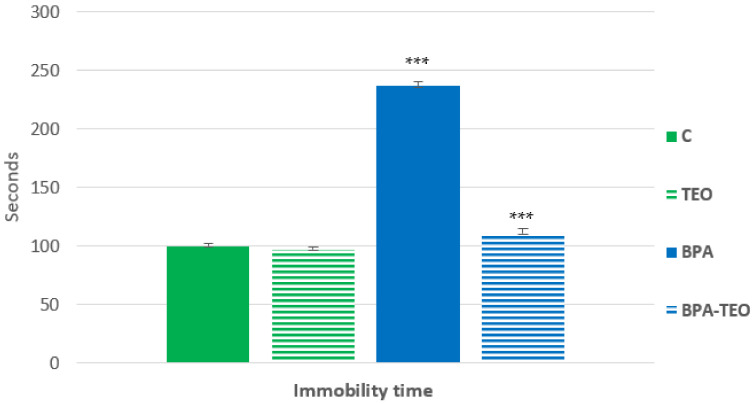
Immobility time during the forced swimming test in control rats (C), control rats treated with TEO (TEO), BPA-intoxicated rats (BPA), and BPA-intoxicated rats treated with TEO (BPA–TEO). Values are presented as mean ± SEM. *** *p* < 0.001 (C vs. BPA); *** *p* < 0.001 (BPA vs. BPA–TEO). *n* = 7 per group. The results are expressed as the mean and standard error of the mean, with ANOVA followed by the Student–Newman–Keuls post hoc test, where significance is defined as *** *p* < 0.001, with analyses performed using Sigma Stat software.

This test measures the immobility time (IMT) conducted on different groups of rats over a 6 min duration. The results showed that the BPA-intoxicated rats (BPA) exhibited a significantly increased immobility time compared with the control rats (C) (*p* < 0.001), which reflects a state of despair. Conversely, a significant decrease (*p* < 0.001) in IMT was observed in the BPA-intoxicated rats treated with TEO (BPA–TEO) compared with the BPA-intoxicated rats (BPA). This suggests that the essential oil (TEO) has the capacity to mitigate the onset of a despair-like behavioral response (Figure 4).

#### 2.2.4. Test for Sucrose Tolerance

**Figure 5:**
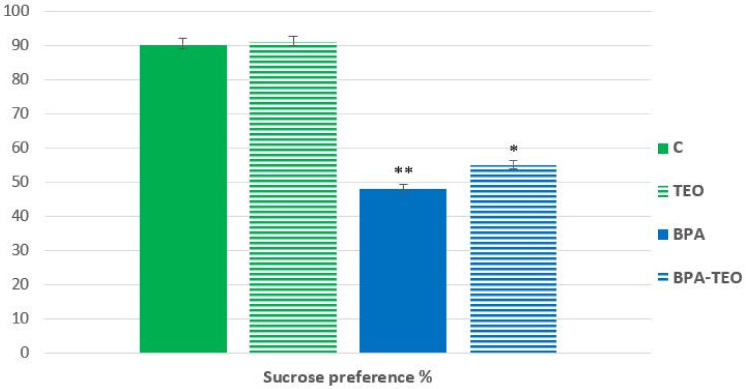
Sucrose preference percentage in control rats (C), control rats treated with TEO (TEO), BPA-intoxicated rats (BPA), and BPA-intoxicated rats treated with TEO (BPA–TEO). Values are presented as mean ± SEM. ** *p* < 0.01 (C vs. BPA); * *p* < 0.05 (BPA vs. BPA–TEO). *n* = 7 per group. The results are expressed as the mean and standard error of the mean, with ANOVA followed by the Student–Newman–Keuls post hoc test, where significance is defined as * *p* ≤ 0.05 and ** *p* < 0.01 with analyses performed using Sigma Stat software.

The test for sucrose tolerance is utilized to assess anhedonia in rodents. Statistical analyses of the test results demonstrated that the sucrose preference of rats exposed to bisphenol A (BPA) differed significantly from that of control rats, wherein it was significantly lower compared with the control group (*p* < 0.01). Furthermore, the administration of TEO to the BPA-exposed rats was found to significantly improve their sucrose preference during the test (*p* < 0.05), which could suggest that TEO essential oil has the ability to mitigate the establishment of anhedonic behavior in this model (Figure 5).

#### 2.2.5. Morris Pool Test

##### Learning Phase

**Figure 6:**
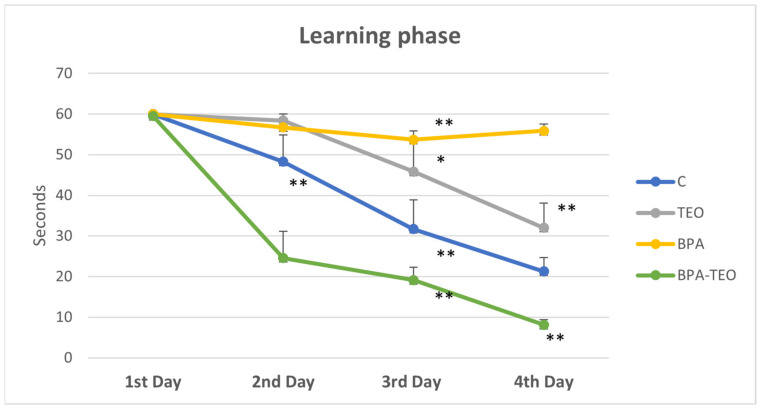
Escape time during the Morris water maze learning phase in control rats (C), control rats treated with TEO (TEO), BPA-intoxicated rats (BPA), and BPA-intoxicated rats treated with TEO (BPA–TEO). Values are expressed as mean ± SEM. Day 2: ** *p* < 0.01 (BPA vs. BPA–TEO). Day 3: (BPA vs. BPA–TEO); ** *p* < 0.01 (C vs. BPA); * *p* < 0.05 (C vs. TEO). Day 4: ** *p* < 0.01 (BPA vs. BPA–TEO); ** *p* < 0.01 (C vs. BPA–TEO). *n* = 7 per group. The results are expressed as the mean and standard error of the mean, with ANOVA followed by the Student–Newman–Keuls post hoc test, where significance is defined as * *p* ≤ 0.05, ** *p* < 0.01, and *** *p* < 0.001, with analyses performed using Sigma Stat software.

The statistical analysis indicated no significant differences among the control groups, BPA-intoxicated rats, and BPA-intoxicated rats treated with TEO on the first day. However, on the second day, the latency time of BPA-intoxicated rats treated with TEO was significantly increased (*p* < 0.01) compared with that of BPA-intoxicated rats. The data demonstrated a significant interaction between essential oil treatment and intoxication regarding latency time, suggesting that the learning patterns of the rats varied based on their intoxication and treatment over the days. Starting from the third day of acquisition, the results revealed that the latency time in intoxicated rats was significantly greater than that observed in both the control rats and intoxicated rats treated with TEO (*p* < 0.01) (Figure 6).

##### Probe Test

**Figure 7:**
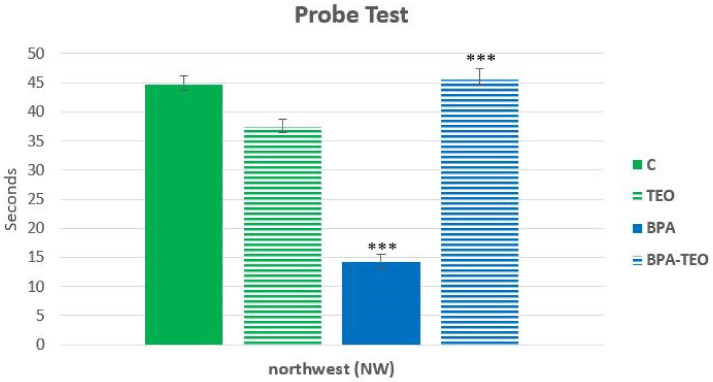
Time spent in the northwest (NW) quadrant during the probe test in control rats (C), control rats treated with TEO (TEO), BPA-intoxicated rats (BPA), and BPA-intoxicated rats treated with TEO (BPA–TEO). Values are expressed as mean ± SEM. *** *p* < 0.001 (C vs. BPA); *** *p* < 0.001 (BPA vs. BPA–TEO). *n* = 7 per group. The results are expressed as the mean and standard error of the mean, with ANOVA followed by the Student–Newman–Keuls post hoc test, where significance is defined as *** *p* < 0.001, with analyses performed using Sigma Stat software.

The statistical analysis revealed a significant reduction in the latency time spent in the northwest (NW) quadrant of the Morris water maze by the BPA-intoxicated rats compared with the control rats (*p* < 0.001). Furthermore, the latency time of the BPA-intoxicated rats treated with TEO was significantly increased compared with the BPA-intoxicated rats (*p* < 0.001) (Figure 7).

**Figure 8:**
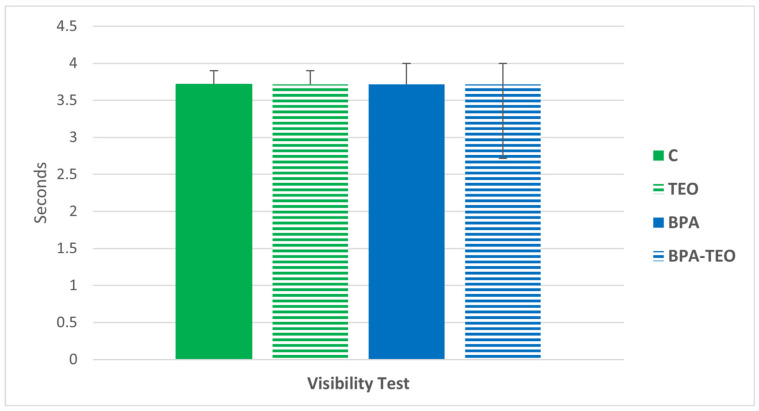
Latency time during the visible platform task in control rats (C), control rats treated with TEO (TEO), BPA-intoxicated rats (BPA), and BPA-intoxicated rats treated with TEO (BPA–TEO). Values are expressed as mean ± SEM. *n* = 7 per group. The results are expressed as the mean and standard error of the mean, with ANOVA followed by the Student–Newman–Keuls post hoc test, with analyses performed using Sigma Stat software.

During the visible platform trial, no significant differences were observed between the different groups (Figure 8).

#### 2.2.6. Open Field Test

**Figure 9:**
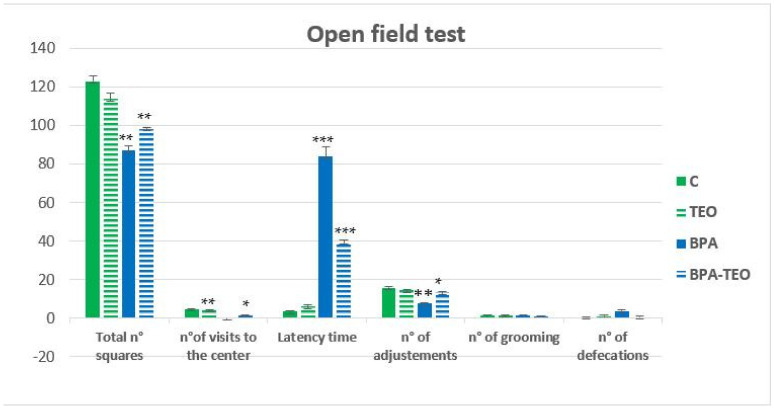
Parameters measured in the open field test across different groups. Data are presented as mean ± SEM. * *p* < 0.05; ** *p* < 0.01; *** *p* < 0.001. *n* = 7 per group. The results are expressed as the mean and standard error of the mean, with ANOVA followed by the Student–Newman–Keuls post hoc test, where significance is defined as * *p* ≤ 0.05, ** *p* < 0.01, and *** *p* < 0.001, with analyses performed using Sigma Stat software.

Statistical analysis of the open field test revealed anxiety-like behavior in BPA-intoxicated rats, characterized by a significant increase in latency time (*p* < 0.001) and a significant reduction in center visits (*p* < 0.01) compared with the control rats. In contrast, treatment with TEO significantly reduced latency time (*p* < 0.001) and increased center visits (*p* < 0.05) in the BPA-intoxicated rats. BPA exposure also impaired locomotor activity, evidenced by fewer squares crossed and fewer adjustments (*p* < 0.01) compared with control rats. However, BPA-intoxicated rats treated with TEO demonstrated a significant improvement, with an increase in squares crossed (*p* < 0.01) and adjustments (*p* < 0.05). No significant differences were observed in defecation or grooming behaviors among the groups. These findings suggest that BPA exposure induced anxiety-like behavior and reduced locomotor activity, effects that were alleviated by TEO treatment (Figure 9).

### 2.3. Biochemical Assay

#### Glucose Levels in the Blood

**Figure 10:**
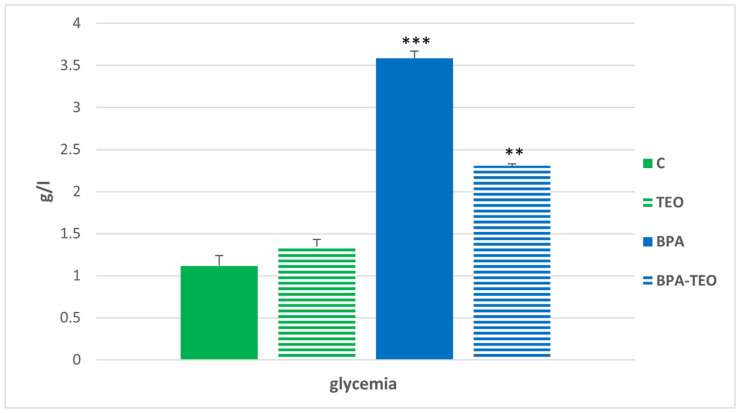
Blood glucose levels in control rats (C), control rats treated with TEO (TEO), BPA-intoxicated rats (BPA), and BPA-intoxicated rats treated with TEO (BPA–TEO). Values are expressed as mean ± SEM. *** *p* < 0.001 (C vs. BPA); ** *p* < 0.01 (BPA vs. BPA–TEO). *n* = 7 per group. The results are expressed as the mean and standard error of the mean, with ANOVA followed by the Student–Newman–Keuls post hoc test, where significance is defined as ** *p* < 0.01 and *** *p* < 0.001, with analyses performed using Sigma Stat software.

The assay results revealed a significant increase in blood glucose levels in BPA-intoxicated rats (*p* < 0.001) compared with the control group. Additionally, a significant decrease in glucose levels (*p* < 0.01) was observed in the BPA-intoxicated rats that received treatment with TEO compared with the untreated intoxicated rats (Figure 10).

### 2.4. Effect of TEO on the Brain Histology of Rats Treated with BPA

**Figure 11:**
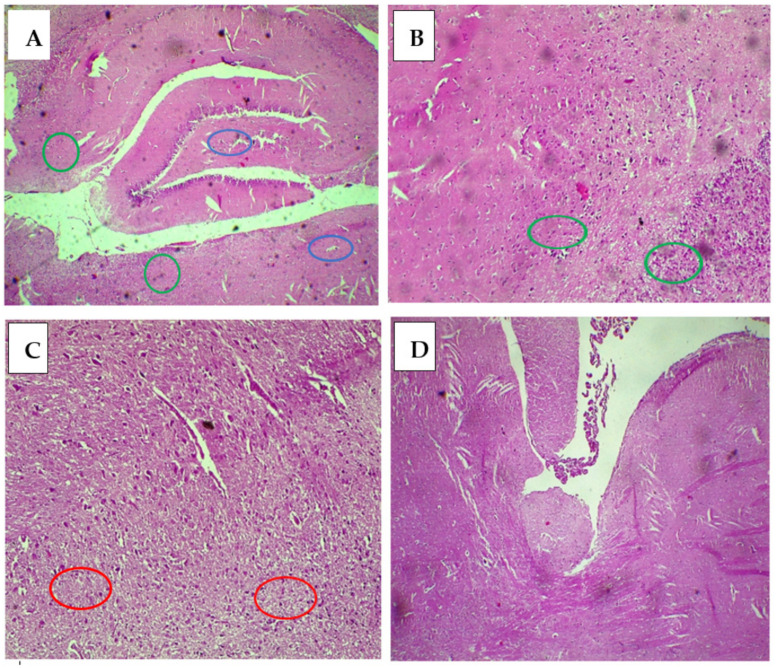
Histological study of the rat brain: (**A**) Optical microscopy of brain tissue stained with hematoxylin and eosin G (×10) in control rats (Control). (**B**) Optical microscopy of brain tissue stained with hematoxylin and eosin G (×10) in controls treated with TEO (TEO). (**C**) Optical microscopy of brain tissue stained with hematoxylin and eosin G (×10) in BPA-intoxicated rats (BPA). (**D**) Optical microscopy of brain tissue stained with hematoxylin and eosin G (×10) in intoxicated rats treated with TEO (BPA–TEO). The results are expressed as the mean and standard error of the mean, with ANOVA followed by the Student–Newman–Keuls post hoc test, with analyses performed using Sigma Stat software.

Microscopic examination of brain tissues revealed several findings following exposure to bisphenol A and treatment with TEO. Control rats (Figure 11A) and those treated with TEO (Figure 11B) exhibited cerebral parenchyma with a normal appearance, characterized by fibrillary tissue (green circle) containing pyramidal and granular neurons as well as cells such as astrocytes, microglia, oligodendrocytes, and endothelial cells (blue circle). In contrast, histological analysis of brain tissues from rats intoxicated with BPA revealed a decrease in neuron density and reactive gliosis (red circle) indicated by an increase in glial cells, specifically astrocytes and oligodendrocytes (Figure 11C). Similarly, animals exposed to BPA and treated with TEO displayed cerebral parenchyma with a subnormal appearance, preserving density but showing moderate focal reactive gliosis (Figure 11D).

### 2.5. In Silico Docking Study

Finding a compound’s binding interaction in the binding pocket of a target protein can be accomplished conveniently via molecular docking. Correlating the in vivo and in silico research is the molecular docking strategy. We discovered binding contacts in all the investigated in vitro targets after docking our examined substances with them for this study [11,19,20]. The virtual analysis of the predominant monoterpene components identified in TEO was conducted within the binding sites of four targets: the human 5HT2C receptor (PDB ID: 8DPH) and monoamine oxidase (MAO) enzyme (PDB ID: 8EEJ), human acetylcholinesterase enzyme (PDB ID: 4EY5), and human butyrylcholinesterase enzyme (PDB ID: 6I0C). The docking results, presented in Table 2 and Figures S1–S4, revealed that most of the components exhibited inhibitory activities against the enzymes, with varying scores that indicated similarities to co-crystallized ligands and root-mean-square deviation (RMSD) < 2.

#### 2.5.1. Interaction with Human 5HT2C Receptor (PDB ID: 8DPH)

Concerning the 5HT2C receptor (VGV isoform, PDB ID: 8DPH), the tested compounds formed high binding affinities (−5.4 to −4.2 Kcal/mol), and they all showed lower binding affinities than T4U = (1R) –8-chloro–1-methyl–2,3,4,5–tetrahydro–1H–3–benzazepine co-crystallized ligand; meanwhile, they showed higher binding affinities than the five controls (four neurotransmitters and one glutamate receptor), which explains the neuroprotective activity of the three *T. ciliatus* essential oils, according to Table 2. Regarding the virtual docking results, thymol and α-terpinene showed the highest binding affinities, with −5.4 and −5.3 Kcal/mol, respectively. Thymol established one conventional H-bond with Cys207 through binding with its hydroxy group; three hydrophobic bonds; Trp130 as a pi–pi stacked bond through interacting with the benzene ring, and Val354 as alkyl and pi–alkyl bonds by interacting with its toluene moiety. Meanwhile, α-terpinene formed only hydrophobic interactions through forming two types of alkyl bonds with Cys207, Ile131, Leu109, Trp130, and Val208. The virtual results of bisphenol A with −7.1 Kcal/mol binding proved that bisphenol A is a highly neurotoxic substance. Meanwhile, serotonin as a neurotransmitter control formed two conventional H-bonds and two pi–alkyl bonds with Ser110, Trp130 and Ile131, and Val208, respectively, with −5.6 Kcal/mol binding energy. There is evidence that the examined compounds promote significant neuro-treatment effects through exhibited strong affinity for the 5HT2C receptor, which showed valuable scores close to the neuro-controls (serotonin and norepinephrine) [21]. All the abovementioned compounds were formed into many Van der Waals interactions in Figure S1.

#### 2.5.2. Interactions with Monoamine Oxidase (MAO) Enzyme (PDB ID: 8EEJ)

With a binding affinity (BA) of −4.6 Kcal/mol, shown in Table 2, thymol is the volatile oil that most closely matches the MAO enzyme’s active site. The formation of many hydrophilic and hydrophobic linkages with substrate cavity residues is explained by this activity [22]. Regarding the monoamine oxidase enzyme, all tested ligands showed binding affinities lower than FAD (co-crystallized ligand; −7.4 Kcal/mol), but they showed the same or close results to the LDP co-crystallized ligand, with −4.8 Kcal/mol.

Regarding the aromatic thymol, it established hydrophilic and hydrophobic interactions with the pocket MAO residues. Interestingly, the phenolic ring of thymol formed two conventional H-bonds with Arg37 and Tyr223; two alkyl bonds with Arg37 and Ile38; together with one with van der Waals interaction with Trp43. α-Terpinene formed only hydrophobic interactions through one pi–sigma bond with Trp43 and five alkyl bonds (alkyl and pi–alkyls) with Arg37, Lys35, and Trp382, along with a van der Waals interaction with Ala34 of −4.4 Kcal/mol. The virtual results of bisphenol A with −5.3 Kcal/mol binding proved that bisphenol A is a high neurotoxic substance (Figure S2). The neurotransmitter controls dopamine and norepinephrine displayed the same binding affinities of the co-crystallized ligand (LDP), −4.7/−4.8 Kcal/mol at the binding cavity with Arg220, Thr44, Trp43 and Arg37, Arg220, Ile38, Thr44, and Trp43 residues, respectively (Figure S2), which confirmed the successive docking process through outputting the same conformation and configuration of these neurotransmitters with the co-crystallized ligand. Finally, the virtual study suggested that thymol, the major compound in TEO, showed activity close to dopamine as a type of MAO isoform substrate or MAO inhibitor, which helps to treat neurodegenerative and neurological illnesses such as depression, Parkinson’s, and Alzheimer’s diseases [23].

#### 2.5.3. Interactions with Human Acetylcholinesterase Enzyme (PDB ID: 4EY5)

All tested components, neurotransmitters, and neurotoxic substances exhibited energy affinities from −5.0 to −7.6 Kcal/mol more than the co-crystalline ligands that established the significant binding positions at the target enzyme cavity in Table 2. Thymol and p-cymene essential oils established BA of −5.8 Kcal/mol, and they formed various pi–hydrophobic bonds, including pi–alkyl bond(s) with Trp286, Tyr72, Tyr341, and Trp286, respectively, and pi–pi stacked bond with Trp286 for both compounds; moreover, thymol formed a conventional H-bond with Ser293 residue. All interactions for all compounds were established together with many Van der Waals interactions in Figure S3. Meanwhile, α-terpinene formed hydrophobic bonds expressed as two pi–sigma bonds with Trp286 and one alkyl bond with the same amino acid with van der Waals interactions of −5.6 Kcal/mol. The virtual results of neurotoxic substances showed higher binding energy with −7.6 Kcal/mol. The neurotransmitter controls serotonin and norepinephrine displayed the highest binding affinities compared with all tested oils and the co-crystallized ligands (NAG and HUP), −6.2 and −6.0 Kcal/mol, respectively, at the binding cavity with various hydrophilic and hydrophobic interactions with many residues (Figure S3).

#### 2.5.4. Interactions with Human Butyrylcholinesterase Enzyme (PDB ID: 6I0C)

Butyrylcholinesterase (BChE), another enzyme of the cholinergic system, is a significant branch of the autonomic system. This study assessed the essential oils tested on BChE activity. All tested substances displaying binding affinities from −4.2 to −6.4 Kcal/mol more than the co-crystalline ligand (GZ5); one of the substances, and all neurotransmitter controls, showed BAs more than the MES. In addition, all essential oils showed BAs less than NAG (co-crystalline ligand), and two neurotransmitters (norepinephrine and serotonin) showed BAs more than the co-crystalline ligand (NAG) that was established in Table 2. Concerning that, thymol formed two pi-attached bonds (one alkyl bond with Ala277 and one donor H-bond with Asn68); on the other hand, α-terpinene established the alkyl bond with the same residue (Ala277) with many Van der Waals interactions, shown (Figure S4).

## 3. Discussion

Bisphenol A (BPA) is an organic compound widely used in the chemical industry. As an endocrine disruptor, BPA poses significant health risks and has been linked to various adverse health effects. Exposure to BPA is primarily believed to occur through ingestion; however, inhalation and skin contact can also play significant roles, particularly in occupational environments. BPA can migrate and leach from metal cans into food and beverages. Once ingested, BPA is absorbed through the digestive system into the bloodstream and subsequently distributed to various tissues throughout the body. In the liver, BPA undergoes conjugation to form bisphenol A glucuronide, a major metabolite that is then excreted in the urine [24]. Due to its lipophilic nature and ability to cross the blood–brain barrier, BPA can influence neuronal functions at various stages and across different species. Numerous studies have investigated the effects of BPA on cognitive functions in rats and mice, utilizing tests such as the Barnes maze, Y-maze, radial arm maze, and Morris water maze. Most of these investigations, particularly those conducted with animal models exposed to low doses of BPA near the current fixed daily limit, have indicated that perinatal, postnatal, prepubertal, and pubertal exposure to BPA may adversely affect learning and memory [25,26].

Aromatic plants generate a wide range of organic compounds with significant ecological and physiological functions. Notably, they synthesize essential oils (EOs) along with secondary metabolites and phenolic compounds. These essential oils can be obtained from different parts of the plant, such as flowers, bark, leaves, roots, and peels. Together with their secondary metabolites and phenolic components, these compounds play vital roles in plant biology and environmental interactions [27,28,29].

Essential oils (EOs) offer many benefits beyond their common usage such as fragrances and flavors, especially concerning their significant role in the therapy of neurodegenerative diseases. The roles of essential oils in reducing disease severity are mediated through various mechanisms, which can differ based on the origin of the oils. Although human studies were not included in this research, there is a strong belief that the presence of essential components could significantly contribute to the prevention and treatment of neurodegenerative disorders [11,30].

This work aims to highlight the risks of BPA exposure in Wistar rats, and its adverse effects on the organisms were evaluated using a neurobehavioral approach. This study also enabled the evaluation of the effectiveness of administering the essential oil of *T. ciliatus* in restoring or mitigating the harm caused by this chemical compound. The essential oil, extracted using the hydro-distillation method, yielded approximately 2.26%. Similarly, Kabouche et al. reported a yield of 2% [29], while Heni et al. (2015) obtained a slightly higher yield of 2.5% [30]. Conversely, Ouakouak et al. observed a moderate yield ranging from 0.84% to 1.53% [31].

Gas chromatography analysis of the *T. ciliatus* essential oil led to the identification of 17 components, which accounted for 98.55% of the total composition, with the major components being thymol (63.33%), p-cymene (13.4%), and σ-terpinene (6.69%). Our results are consistent with the work of many previous studies [32,33], which found that thymol is the main compound in the essence of *T. ciliatus*, followed by p-cymene and σ-terpinene.

Our data are similar to that of other authors, except for certain variations in proportions, which are mainly due to regional differences, climatic conditions, soil physicochemical factors, the part of the plant used, the plant’s age, the stage of the vegetative cycle, or even genetic factors [34,35].

After 60 days of BPA exposure, significant deficits in spatial memory were observed in rats using the Morris water maze. The BPA-exposed rats took much longer to locate the platform, particularly during the final two days of the acquisition phase, compared with the control group. Additionally, these rats spent significantly less time in the northwest quadrant than the control rats. Results from the Y-maze test also revealed a marked decline in memory performance in the BPA-exposed rats relative to the controls. These findings suggest that BPA exposure negatively impacts rat behavior, impairing both learning and memory.

Numerous studies have indicated that BPA exposure can disrupt learning and memory in mice. For example, Carr et al. (2003) found that oral administration of low (100 mg/kg) and high (250 mg/kg) doses of BPA from postnatal days 1 to 14 altered gender-dependent patterns of spatial information acquisition (at the low dose) and spatial memory retention (at the high dose) in mice [36]. Additionally, Xu and colleagues (2010) reported that perinatal maternal exposure to BPA in ICR mice at doses of 0.5, 5, and 50 mg/kg/day significantly increased escape latency in the Morris water maze. Exposure to BPA at 0.5 or 5 mg/kg/day also reduced the percentage of time spent in the quadrant where the platform was previously located, both at postnatal day (PND) 21 and PND 56. The passive avoidance test showed that BPA-exposed mice made more errors when stepping down from a platform after receiving a foot shock, indicating memory impairments [37].

In another study, Tian et al. (2010) found that mice treated with BPA at doses of 100 and 500 mg/kg/day exhibited reduced alternation behavior in the Y-maze test, indicating impairments in working memory at both dose levels. Additionally, BPA-treated mice demonstrated deficits in novel object recognition, as evidenced by decreased central locomotion and a lower frequency of visits to the central zone during test [38].

A study on rats exposed to BPA during the juvenile stage found that males receiving high doses exhibited deficits in spatial memory and increased anxiety-like behavior. These impairments were associated with a downregulation of the NMDA receptor subunit NR2, the AMPA receptor subunit GluR1, and a reduction in pyramidal neuron spine density in the hippocampus [39]. Additionally, perinatal exposure to BPA at doses of 50, 5, and 0.5 mg/kg/day significantly inhibited the expression of the NMDA receptor subunits NR1, NR2A, and NR2B in the hippocampus of developing mice, particularly at postnatal day 56 [37].

Bisphenol A (BPA) affects spatial memory in rats through several interconnected molecular mechanisms that disrupt neurodevelopment and cognitive function. One primary mechanism is endocrine disruption, where BPA mimics estrogen and interferes with hormonal signaling, particularly affecting estrogen receptors in the hippocampus, a critical area for spatial memory. This disruption can impair synaptic plasticity, which is essential for memory formation [40]. Additionally, BPA exposure leads to increased oxidative stress, resulting in the generation of reactive oxygen species (ROS) that can damage neuronal cells and disrupt cellular signaling pathways, further contributing to cognitive deficits [2]. Neuroinflammation is another significant factor; BPA can activate microglia and promote the release of pro-inflammatory cytokines, which are associated with cognitive decline and impaired synaptic function [3]. Furthermore, BPA may alter neurotransmitter systems, particularly the balance between glutamate and GABA, both of which are crucial for synaptic plasticity and memory. BPA exposure also hinders neurogenesis in the hippocampus, limiting the brain’s capacity for learning and memory. Lastly, BPA can induce epigenetic modifications such as changes in DNA methylation, which affect gene expression related to neurodevelopment and synaptic function, ultimately impacting spatial memory capabilities. Collectively, these mechanisms illustrate how BPA exposure can lead to significant cognitive impairments in spatial memory.

The results from the forced swimming test (FST) indicate that intoxicated rats exhibit a significantly longer immobility time (IMT) compared with control rats, which display minimal immobility. Similarly, findings from the dark/light test reveal that while control and sham-treated control rats prefer the dark compartment, intoxicated rats spend significantly more time in the illuminated section. In the open field test, untreated intoxicated rats demonstrate a longer latency period and fewer visits to the center compared with control rats, sham-treated controls, and intoxicated rats that received treatment. Furthermore, statistical analyses indicate that BPA-exposed rats exhibit a significantly lower sucrose preference compared with the control group, suggesting potential anhedonia.

Extensive research has demonstrated that exposure to this endocrine-disrupting chemical (EDC) results in heightened anxiety and depressive behaviors in rats. For instance, in the well-established predator odor stress paradigm, subjects exposed to bisphenol A (BPA) exhibited a significantly exacerbated avoidance response to an aversive stimulus (fox odor) compared with vehicle-treated control subjects. This heightened avoidance behavior suggests that BPA-exposed animals displayed enhanced levels of anxiety relative to the control group [41]. The predator odor stress test is a widely used animal model for assessing anxiety-related behaviors, as the presence of a predatory scent elicits robust fear and avoidance responses in rodents. The observed exaggerated avoidance of the fox odor cue by the BPA-exposed rats indicates that exposure to this EDC led to a marked increase in anxiety-like responses in both male and female animals. These findings contribute to the growing body of evidence that BPA, as an endocrine disruptor, can adversely impact emotional regulation and stress reactivity, leading to the manifestation of enhanced anxiety-like and depressive-like behaviors in laboratory rodents. The heightened aversive response to the predatory odor stimulus further highlights the deleterious effects of BPA on the neurobiological pathways involved in fear, anxiety, and emotional processing [41]. Studies have shown that exposure to this endocrine-disrupting chemical (EDC) results in increased anxiety-like and depressive-like behaviors in both male and female rats. For example, in the predator odor stress paradigm, subjects exposed to bisphenol A (BPA) exhibited exacerbated avoidance of an aversive fox odor stimulus compared with controls, suggesting enhanced anxiety levels. Furthermore, pubertal male rats given high-dose BPA (40 mg/kg/day) displayed an anxiogenic profile on the elevated plus maze, with fewer open arm entries. This effect was not observed in females at the same dose [42]. Gestational and early-life exposure to lower BPA doses (2 mg/kg/day) also led to increased anxiety and depressive-like behaviors in the adult offspring of exposed dams. These findings demonstrate the sex-dependent and developmental stage-specific impacts of BPA on emotional behaviors in laboratory rodents [43,44]. Intriguingly, the behavioral consequences of BPA exposure exhibited a distinct sex-dependent pattern. Specifically, female rats displayed a more pronounced anxiety-like profile compared with their male counterparts, whereas male rats exhibited a more prominent depressive-like phenotype relative to control subjects [45,46]. Anxiogenic-like effects, such as reduced open arm exploration in the elevated plus maze, have been observed in studies using the mouse version of this behavioral paradigm [47]. Additionally, several studies have reported increased immobility in BPA-exposed animals, a key indicator of depressive-like behavior [44,48,49]. This behavioral measure, typically assessed using the forced swim test or tail suspension test, reflects a state of behavioral despair or learned helplessness, which is characteristic of depressive-like phenotypes in animal models. Interestingly, some studies suggest that while BPA administration may not directly alter immobility time, it can eliminate the sex differences observed in control rodents [50].

The impact of bisphenol A (BPA) exposure varies significantly depending on the stage of development. During the perinatal period, which includes utero exposure and the early postnatal phase, BPA can disrupt critical developmental processes. Studies have shown that perinatal BPA exposure can lead to reproductive system abnormalities, neurodevelopmental impairments, and an increased risk of metabolic disorders later in life. For instance, exposure to BPA during pregnancy has been linked to adverse reproductive effects in male offspring, emphasizing the heightened vulnerability of developing systems to endocrine disruptors [50]. In the postnatal period, BPA exposure remains a concern as the brain and other organs continue to mature. Research indicates that exposure during this stage can impair cognitive functions, influence behavior, and disrupt immune system development. For example, postnatal BPA exposure has been associated with anxiety-like behaviors and deficits in learning and memory in animal models [51]. During puberty, a critical period of hormonal changes, BPA can disrupt hormonal signaling, potentially impairing sexual maturation and reproductive health. Its estrogenic activity may alter these processes, resulting in long-term reproductive consequences. In contrast, BPA exposure in adulthood tends to exacerbate preexisting health issues rather than cause developmental disruptions. In adults, BPA exposure has been linked to an increased risk of metabolic syndrome, cardiovascular diseases, and certain cancers. However, the developmental effects observed in younger populations are less pronounced in adults [51,52,53].

The treatment with TEO, administered intraperitoneally at a dose of 0.1 mL/kg/day, significantly improved performance across various neurobehavioral tests. These findings suggest that *T. ciliatus* effectively attenuated anxiety, depression, anhedonia, and hypoactivity while enhancing learning abilities as well as spatial and working memory in previously intoxicated rats.

*Thymus ciliatus* essential oil may enhance spatial memory and reduce anxiety in rats through several key biochemical mechanisms. One primary mechanism is its modulation of neurotransmitters, particularly serotonin and gamma-aminobutyric acid (GABA). Increased GABAergic activity can lead to reduced anxiety, while serotonin plays a crucial role in mood regulation and cognitive functions. Additionally, the essential oil possesses significant antioxidant properties, which help mitigate oxidative stress in the brain, a factor closely linked to cognitive decline and anxiety disorders. By reducing oxidative damage, the oil may contribute to improved memory and emotional stability [54]. Furthermore, *T. ciliatus* essential oil exhibits anti-inflammatory effects that protect neuronal health. Neuroinflammation is strongly associated with cognitive impairments and mood disorders; therefore, reducing inflammation could support better cognitive function and lower anxiety levels [55]. Another potential mechanism is the enhancement of cholinergic activity, as increased acetylcholine levels are known to improve spatial memory performance in rats [56].

Thymol is recognized as one of the primary bioactive compounds found in various *Thymus* (thyme) species. It exhibits a broad spectrum of pharmacological properties, including free radical scavenging, antioxidant, anti-inflammatory, antibacterial, antispasmodic, analgesic, antiseptic, antifungal, and antitumor activities [50,51,52,53]. Thymol plays a crucial role in enhancing overall health and immunity, and studies suggest it may help alleviate symptoms associated with various disorders in both animals and humans [57]. Previous research has indicated that thymol can mitigate depression-like symptoms and increase brain-derived neurotrophic factor (BDNF) levels in the hippocampus in an animal model of corticosterone-induced depression [58]. Additionally, studies have reported that thymol administration leads to an upregulation of neurotransmitters and the inhibition of pro-inflammatory cytokines in an animal model of chronic mild stress [59]. These findings suggest that thymol exerts beneficial effects on mood regulation and neuroinflammation in depression. In the current study, pre-administration of thymol resulted in significantly higher BDNF levels compared with rats exposed to cadmium, indicating that thymol contributes to hippocampal function regulation by enhancing BDNF expression and promoting brain plasticity [60]. Previous studies have also highlighted the neuroprotective properties of *T. ciliatus* extracts. Notably, it was found to mitigate oxidative stress and prevent hippocampal neuronal damage following transient global cerebral ischemia in rats. Moreover, *Thymus vulgaris* (common thyme) has been shown to enhance antioxidant capacity in serum and brain tissues while reducing malondialdehyde (MDA) levels. However, further research is required to fully elucidate the underlying mechanisms of its neuroprotective effects [61].

Thymol, a major bioactive compound in *Thymus ciliatus* essential oil, plays a crucial role in enhancing brain plasticity and mitigating the neuronal damage induced by bisphenol A (BPA). One of its primary mechanisms of action is its potent antioxidant activity, which helps neutralize reactive oxygen species (ROS) generated by BPA exposure. By reducing oxidative stress, thymol protects neuronal cells from damage and supports cellular integrity, an essential factor in maintaining brain plasticity [62]. In addition to its antioxidant effects, thymol exhibits strong anti-inflammatory properties that help counteract BPA-induced neuroinflammation. Chronic inflammation in the brain is known to impair neurogenesis and synaptic plasticity, leading to cognitive deficits and memory impairment. By modulating inflammatory pathways, thymol may support neuronal function and improve cognitive performance [55].

Moreover, thymol exhibits neuroprotective properties that enhance neuronal survival and function by modulating various signaling pathways involved in cell survival and apoptosis. This neuroprotective effect is especially important in counteracting the neurotoxic impact of bisphenol A (BPA), which can lead to neuronal death and cognitive deficits [63]. Research also suggests that thymol stimulates neurogenesis, particularly in the hippocampus, a region crucial for learning and memory. By promoting the growth of new neurons and synaptic connections, thymol contributes to enhanced brain plasticity. Furthermore, thymol may increase cholinergic activity, which plays a pivotal role in learning and memory processes. Elevated acetylcholine levels are associated with improved cognitive performance and memory retention, counteracting the cognitive impairments linked to BPA exposure. In summary, thymol from *T. ciliatus* essential oil serves a multifaceted role in enhancing brain plasticity and protecting against BPA-induced neuronal damage through its antioxidant, anti-inflammatory, neuroprotective, and neurogenic properties. Additionally, exposure to bisphenol A (BPA) significantly increased blood glucose levels in adult rats, mirroring previous studies in which elevated blood glucose levels were observed in adult mice following BPA exposure. For instance, a subcutaneous injection of 100 mg/kg BPA resulted in hyperglycemia in adult mice as well as insulin resistance, which was similarly reported in male mouse offspring exposed to 10 mg/kg/day BPA during gestation [59,60]. These findings underscore the heightened sensitivity of the endocrine pancreas to BPA, a known endocrine disruptor. At concentrations as low as 1 nM, BPA can trigger increased [Ca^2+^] oscillations in β cells, activating the CREB transcription factor. Additionally, BPA disrupts low-glucose-induced [Ca^2+^] oscillations in glucagon-releasing α-cells. Importantly, the concentrations at which BPA affects these cells are within the range found in human serum (0.9–8.8 nM) [61]. *In vivo* studies have shown a rapid increase in plasma insulin and a corresponding decrease in blood glucose levels at doses as low as 10 µg/kg. Prolonged exposure to 100 µg/kg/day BPA altered pancreatic insulin content and secretion, leading to postprandial hyperinsulinemia and insulin resistance in male mice [64]. BPA, along with other endocrine disruptors such as persistent organic pollutants and phthalates, may contribute to the exacerbation of type II diabetes progression [65,66,67,68].

In contrast, the results for blood glucose levels in animals exposed to BPA and treated with TEO show a reduction in glucose levels compared with the BPA-intoxicated rats. These results align with earlier studies highlighting the antihyperglycemic effects of other Thymus species. In this context, the antihyperglycemic effects of the aqueous extract and essential oil of the plant may be attributed to the high concentration of secondary metabolites, particularly the isomeric phenolic monoterpenes thymol and carvacrol, found in the essential oil [69].

The administration of bisphenol A (BPA) to rats has been associated with significant histological damage in the brain, consistent with existing research that highlights BPA’s neurotoxic effects. As an endocrine disruptor, BPA can adversely affect both brain function and structure. Studies have shown that BPA exposure leads to neuronal cell death, glial activation, and alterations in brain morphology, particularly in areas such as the hippocampus, which plays a critical role in learning and memory. The oxidative stress induced by BPA results in elevated levels of reactive oxygen species (ROS), contributing to neuronal damage and inflammation in the brain [9]. While the correction of histological damage was modest, these findings suggest that *T. ciliatus* essential oil may possess therapeutic potential in mitigating some of the neurotoxic effects of BPA. This aligns with previous studies showing that essential oils can positively influence neurobehavioral outcomes and enhance cognitive function in animal models [55].

Finding a compound’s binding interaction in the binding pocket of a target protein can be accomplished conveniently via molecular docking. Correlating the in vivo and in silico research is the molecular docking strategy. The study of the promising monoterpene components recognized from the essential oils of thyme was conducted within the binding sites of the human 5-HT2C receptor and three enzymes: monoamine oxidase (MAO), human acetylcholinesterase, and human butyrylcholinesterase.

Serotonin (5-HT) and dopamine (DA) neurotransmitters are closely related in both healthy and pathological brain functioning. The majority of neuropsychiatric drugs work in these two systems, and their disruption affects mental states. Understanding the interplay of the transmitter systems is crucial for comprehending psychopathology, medication, and normal brain function. Right now, serotonin (5-HT) selective reuptake inhibitors (SSRIs) that inhibit the 5-HT transporter (5-HTT) are the medications most often prescribed for mental health issues to treat anxiety and depression. In addition to being given as the initial course of treatment for depressive disorders, they are also administrated for a wide range of other psychiatric conditions, such as bipolar, generalized anxiety, panic, and obsessive-compulsive (OCD) disorders. SSRIs have problems with both efficacy and negative effects while being used widely [70].

MAO enzymes, especially MAO-B, are physiologically involved in the recycling of dopamine, a crucial neurotransmitter. It is known that inhibitors of the enzymes that metabolize dopamine can prevent Alzheimer’s disease (AD), oxidative stress, and Parkinson’s disease [19]. The level of MAO-B enzyme is three times more elevated in cases of brain disorders. Consequently, molecular docking is being conducted on MAO-B selective inhibition, particularly with respect to plant-based sources, in order to effectively control the brain damage. Thus, our tested compounds, owing to their considerable efficacy against MAO enzymes, are of the same significance of neurotransmitters as target agents against brain damage.

In addition, an essential neurotransmitter involved in the transmission of impulses across synapses and toward the effector organ is acetylcholine (ACh). ACh activates its receptors after release from the pre-synaptic nerve terminal, sending a powerful impulse to the post-synaptic neuron. These esterases metabolize and recycle ACh within a pre-synaptic nerve terminal, thereby stopping its function. Because of the selective degeneration of cholinergic neurons in brain disorders like AD, there is a significant ACh deficiency. This makes it harder to consolidate memories and carry out daily tasks. Inhibiting the esterases that metabolize ACh is one treatment strategy that can be used to make the ACh released stay at the nerve terminal longer and provide a stronger reaction. Since they are now the only successful therapy approach among the currently available anti-AD medications, physicians mostly rely on these acetylcholinesterase inhibitors, such as galantamine (alkaloid) and rivastigmine (polyphenol), that have the ability to scavenge free radicals, which lowers oxidative stress, and they subsequently decrease neurodegeneration and inflammatory processes that are mediated by free radicals [22].

Regarding the virtual results for the four neuro-targets, these findings support the obtained in vivo results. Thymol, which is found only in *T. ciliatus*, was compared with other substances and neuro-controls, indicating that it appears to be one of the most potent neuroprotective substances, dependent on the subclasses of terpenes present in thyme, as revealed in all the examined receptors. The docking results, recorded in Table 2 and Figures S1–S4, revealed that thymol, as the major compound in TEO, exhibited inhibitory activities against the most tested in silico targets, the MAO-B, AChE, and BChE enzymes, with varying scores from −4.6 to −5.8 Kcal/mol, that demonstrated similarities to MES (−4.6 Kcal/Mol), a co-crystallized ligand of BChE enzyme; slightly less than LDP (−4.8 Kcal/Mol), a co-crystallized ligand of MAO-B enzyme; or slightly exceeded NAG and HUP with −5.0 and −4.5 Kcal/Mol, respectively, co-crystallized ligands of AChE enzyme.

Continuing the analysis, in vivo studies in animal models, which were well established and discussed previously in this manuscript, were conducted to assess the stability and substrate performance of the molecular interactions for the inhibitors. Evidence suggests that these substances have the ability to inhibit serotonin, monoamine oxidase, and esterase enzymes, indicating their potential utility against multiple pathogenic targets and suggesting they may serve as an initial treatment for brain disorders like Alzheimer’s disease. Interestingly, the results showed that all tested essential oils were more potent acetylcholinesterase (AChE) inhibitors than butyrylcholinesterase (BChE) inhibitors.

## 4. Materials and Methods

### 4.1. Thymus ciliatus Extraction and Identification by GC-MS

*Thymus ciliatus* was collected in May 2023 from the Saida province, specifically in Oued El-Kifeh, Tifrit, Saida, Algeria. The plant was harvested at its full flowering stage. Identification of the plant was carried out by a taxonomic expert (Professor Hasnaoui Okkacha).

The essential oil was extracted through hydro-distillation. The ground plant material was mixed with distilled water and heated to boiling. A separatory funnel was then employed to separate the essential oil from the water based on their differences in density [71].

The essential oil was analyzed at the Natural Molecules Chemistry Laboratory at Gembloux, University of Liège, Belgium, using gas chromatography (Agilent 7890 system) coupled with a mass spectrometer (Agilent 5975; Agilent Technologies Inc., California (Santa Clara, CA, USA). The analysis was performed using an HP5 MS column (30 m × 0.25 mm; 0.25 μm). The oven temperature was initially held at 50 °C for 3.2 min and then raised to 300 °C at a rate of 8 °C/min. The injector temperature was set to 280 °C. Ionization was achieved using 70 eV electron impact, with the electron multiplier set to 2200 eV. The ion source temperature was maintained at 230 °C, and mass spectral data were collected in scan mode from 33 to 450 *m*/*z*. The carrier gas, helium, was used at a flow rate of 0.9 mL/min.

### 4.2. Animal Treatment and Dividing Groups

The experiments were carried out on young male Wistar rats (at the age of two months) housed in the animal facility of the Department of Biology at the University Dr. Tahar MOULAY, Saida, Algeria. The rats were individually caged, with seven rats per group, and kept in a well-ventilated room maintained at a temperature of 21 °C ± 1 °C under artificial lighting to simulate a day/night cycle. The animals had unrestricted access to food and water. All efforts were made to minimize distress to the animals, in accordance with the guidelines outlined in the European Council Directive (86/609/EEC).

BPA intoxication was administered orally at a dose of 50 mg/kg to young rats [71]. After 60 days of BPA exposure, the intoxicated and control groups were treated with *T. ciliatus* essential oil (TEO) at a dose of 0.1 mL/kg for 21 days via the intraperitoneal route [72]. The rats were divided into four groups: Group C (control), which received distilled water only; Group TEO, which received distilled water and TEO treatment; Group BPA, which received the BPA oral solution; and Group BPA–TEO, which received BPA and TEO treatment.

### 4.3. Neurobehavioral Tests

A variety of neurobehavioral parameters were assessed, including locomotor activity, anxiety and depression levels, and learning and memory abilities, using several testing paradigms such as the dark/light, forced swimming, open field, Morris maze, sucrose preference, and Y-maze tests. To eliminate any potential olfactory cues that could influence the results, all apparatuses were cleaned with an ethanol solution (70–90%) after each animal’s trial. The entire sequence of tests was filmed and recorded for subsequent analysis of the behavioral variables [72].

#### 4.3.1. Dark/Light Test

In the dark/light compartment test, researchers assessed anxiety levels in rats by observing their preference for the darker compartment. The apparatus consists of a two-compartment box (22 cm × 16 cm × 23 cm) with a small opening (6 cm × 5 cm) between a dark and a brightly lit area. After being placed in a dark compartment, each rat explored the environment for 3 min. Following this, the time spent in each compartment during a 5 min period is measured, allowing for a comparison of exploratory behavior and an evaluation of anxiety levels in the animals [73].

#### 4.3.2. Y-Maze Test

The Y-maze is designed with three identical arms arranged in an equilateral triangle, each measuring 50 cm long, 10 cm wide, and 20 cm high. This test is used to evaluate working memory in rodents. A rat is placed in one arm, facing the intersection, and is allowed to explore for 5 min. A branch is considered entered when all four paws are inside. The sequence and total number of visits to each arm are recorded to assess general activity and visit distribution. Data analysis includes calculating alternations as a percentage using the formula: Number of alternations/(Number of visits − 2) × 100 [74].

#### 4.3.3. Test of Forced Swimming

This test is designed to assess a state of resignation, which is indicative of depressive behavior in rodents, as shown by significant immobility, as originally defined by its creator. The experimental setup consists of a transparent Plexiglas cylinder, measuring 20.7 × 39 cm in diameter and height, respectively, filled three-quarters full of water at a temperature of 22 ± 1 °C. In the forced swim test, rats are exposed to the environment for 6 min. The measured parameters include the time spent moving, during which the animal swims actively using all four paws, and the time spent immobile, during which the animal floats with minimal movement. This immobility is considered a sign of behavioral despair [75].

#### 4.3.4. Test for Sucrose Tolerance

The test for sucrose tolerance is a widely recognized behavioral assessment used to evaluate anhedonia, a significant symptom of depression, in rodent models. This test involves two phases: first, a habituation phase where the animal is acclimated to drinking from two identical bottles, one containing tap water and the other a sucrose solution at a concentration typically ranging from 1% to 4%. This initial phase allows the rodents to familiarize themselves with the experimental setup and encourages drinking from both sources. Following habituation, the preference test phase commences, during which the animals are given a choice between tap water and the sucrose solution. Their preference is measured by comparing the consumption of the sucrose solution to the total fluid intake over a specified period, typically one to four hours. The sucrose tolerance is determined using the following formula: Sucrose preference (%) = (Sucrose solution consumption/Total liquid consumption) × 100 [76].

#### 4.3.5. The Morris Pool

The test evaluates spatial learning, orientation, and visual–motor guidance using a 160 cm diameter, 60 cm high opaque pool filled with water to a depth of 30 cm at 22–23 °C. A platform, 10 cm in diameter and 28 cm high, is submerged 2 cm below the surface in the NW quadrant. The experiment lasts four days, with four trials each day, where rats are placed facing different cardinal points for 60 s to find the platform. If unsuccessful, they are guided to it and remain there for 20 s. On the fifth day, a probe test occurs with the platform removed, measuring the time spent in the NW quadrant during a 60 s trial. Following a 2 h delay, a visible platform phase includes four trials, each lasting up to 60 s with 40 min intervals [77].

#### 4.3.6. Open Field Test

The “test open field” was an open-topped rectangular wooden enclosure measuring 90 × 70 × 60 cm, brightly lit, with a dark background marked by lines delimiting 10 × 10 cm squares. The rat was first placed in one of the four corners, with its head facing the corner. Its behavior was observed for 5 min. The following parameters were evaluated: latency time, number of squares crossed by the rat, visits to the center, adjustments, grooming episodes, and defecations. This test evaluates the exploratory behavior of the rat in a stressful environment. The number of squares crossed and matings indicates the exploratory activity and emotional state of the rat, while the other parameters give an idea of its emotional well-being [78].

### 4.4. Biochemical Assays

At the end of the experimentation period, blood was collected from the animals through cardiac puncture. The volume of blood collected per animal typically ranged from approximately 1 to 2 mL.

#### Blood Glucose Levels

Blood glucose levels were assessed using a colorimetric method with reagents from Cypress Diagnostics. This technique involves glucose oxidase (GOD) facilitating the oxidation of glucose to gluconic acid, which generates hydrogen peroxide (H_2_O_2_). The H_2_O_2_ is then detected through a reaction with phenolaminophenazone in the presence of peroxidase (POD). The intensity of the resulting color correlates directly with the glucose concentration in the sample, measured spectrophotometrically at 510 nm.

### 4.5. A Study of Brain Histology

The animals were sacrificed at the end of the experimentation. The body of the animal was opened, and the organs (brains) were carefully extracted, rinsed with cold physiological saline (0.9% NaCl), dried, and then fixed in 1/10 formalin for the purpose of studying them later using histological techniques. These tissues underwent dehydration using graded alcohol solutions before being processed and included in wax paraffin. Next, sections were cut to a thickness of 3 μm using a microtome, and the resulting sections were placed on slides and stained with hematoxylin and eosin. Finally, the slides were examined by light microscopy [79].

### 4.6. In Silico Docking Study

In silico analysis was conducted on three essential oils—thymol, p-cymene, and α-terpinene—extracted from *T. ciliatus*. This study also included one neurotoxic, bisphenol A, and four controls (selected neurotransmitters): serotonin, norepinephrine, glutamic acid, and dopamine, and one glutamate receptor (N-methyl-D-aspartic acid). The SMILES representations of these compounds were downloaded, and the protocol was validated as described in references [20,79]. Four neuro-targets were investigated: the human 5HT2C receptor (PDB ID: 8DPH, 3.20 Å; VGV isoform) [80], monoamine oxidase (MAO) (PDB ID: 8EEJ, 1.54 Å) [80], human acetylcholinesterase receptor (PDB ID: 4EY5, 2.30 Å) [70,80], and human butyrylcholinesterase receptor (PDB ID: 6I0C, 2.76 Å) [80]. These targets were sourced from the Protein Data Bank (PDB) at https://www.rcsb.org, accessed on 11 June 2024 and 4 July 2024. The in-silico analysis was achieved by PyRx Auto dock Vina (Scripps Research, La Jolla, CA, USA), following previously published methodologies [11,19,20,80].

### 4.7. Statistical Analysis

The results are presented as the mean (M) of individual values, with the standard error of the mean (SEM). To compare multiple means, an analysis of variance (ANOVA) was accomplished, incorporating the intoxication factor (BPA, C) and/or the treatment factor (solvent, TEO). Subsequently, the Student–Newman–Keuls post hoc test was applied. Significance levels were defined as follows: * *p* ≤ 0.05 indicates a significant difference, ** *p* < 0.01 denotes a very significant difference, and *** *p* < 0.001 represents a highly significant difference compared with the controls. Statistical analyses were conducted using Sigma Stat software.

## 5. Conclusions

The findings of this study indicated that exposure to bisphenol A in young male Wistar rats resulted in a deficient neurobehavioral state. However, treatment with *Thymus ciliatus* essential oil was effective in mitigating these alterations and improving the neurobehavioral impairments in the rats. These positive effects are further supported by the results from the silico docking study analyses.

## Figures and Tables

**Table 1:** Chemical composition of *Thymus ciliatus* essential oil (TEO).

Identified Compounds	RT	RI^b^	RI^a^	TEO%
endo-Borneol	12.8956	1165	1165	0.4
σ-Cadinene	22.8601	1464	1502	0.2
Caryophyllene	20.1832	1412	1415	2.5
Caryophyllene oxide	24.4181	1494	1578	1.09
Copaene	18.9672	1377	1377	0.13
p-Cymene	8.8085	1024	1024	13.4
Diphenylamine	25.4695	1532	1622	0.59
Humulene	21.0783	1429	1442	0.09
Linalool	10.9365	1098	1099	0.07
Lupanine	38.1531	2142	2159	3.4
Multiflorine	41.2016	2317	2320	2
α-Pinene	6.33	930	931	2.26
β-Pinene	7.8078	988	981	1.4
L-α-Terpineol	13.8414	1197	1192	0.1
σ-Terpinene	9.7627	1057	1056	6.69
Thymol	16.8814	1302	1302	63.33
Thymyl methyl ether	14.9131	1234	1235	0.9
Total identified (%)				98.55

RT is retention time in minutes; RI_a_ refers to the retention index value reported in the scientific literature; RI_b_ represents the retention index value that was experimentally determined on an HP-5 capillary column using the standard series of n-alkane compounds ranging from C_8_ to C_30_.

**Table 2:** Docking investigation of the essentials shown in *Thymus ciliatus* against neuro-enzymes.

Proteins	8DPH			8EEJ			4EY5			6I0C		
Ligands	BE	BN	AAs	BE	BN	AAs	BE	BN	AAs	BE	BN	AAs
Co-Crystallized Ligand(s)												
T4U	−6.1	5 *	Asn331, Asp134, Cys207, Ile131, Leu209, Leu350, Phe327, Tyr358, Val208, Val354									
LDP				−4.8	4	Arg37, Arg220, Ile38, Thr44, Trp43, Tyr223						
FAD				−7.4	5	Arg37, Asp36, Asp224, His375, Ile38, Lys35, Thr44, Trp43, Tyr223						
NAG							−5.0	2	Arg296, Glu292, Gln291, Leu289, Leu76, Trp286, Ser293, Try72, Val294, Tyr341	−5.1	3	Asn289, Asn68, Ala277, Asp70, Gly283, Gln119, Thr284, Ile69, Pro285, Val280, Ser287, Thr120
HUP							−4.5	2	Arg296, Gly342, Glu292, Ser293, Leu289, Try341, Phe295, Trp286, Val294, Tyr72			
MES										−4.6	3 *	Asn289, Glu276, Ala277, Gln119, Thr284, Ser287, Gly283, Val280, Asn68
GZ5										−3.0	5	Ala277, Asp70, Asn68, Asn289, Asn83, Ile69, Gln119, Gly283, Pro285, Thr284, Ser72, Ser79, Ser287, Pro84, Thr120, Trp82, Val280, Tyr332
Compounds												
α-Terpinene	−5.3	3	Asn331, Cys207, Ile131, Leu209, Phe327, Trp130, Val208, Val215	−4.4	4	Arg37, Ala34, Lys35, Trp43, Trp382	−5.6	3	Leu289, Tyr341, Ser293, Tyr72, Trp286	−4.4	2	Ala277, Asn68, Asn289, Gln119, Glu276, Thr120
p-Cymene	−5.1	4	Asn331, Cys207, Ile131, Leu209, Trp130, Val208, Val215	−4.3	3	Arg37, Lys35, Trp43, Trp382	−5.8	3	Leu289, Tyr341, Ser293, Tyr72, Trp286	−4.2	1	Ala277, Asn68, Asn289, Asp70, Gln119, Glu276, Thr120
Thymol	−5.4	5	Asp134, Cys207, Ile131, Leu209, Ser110, Trp130, Tyr358, Val209, Val354	−4.6	4	Arg37, Ile38, Trp43, Tyr223	−5.8	4	Arg296, Tyr341, Glu292, Ser293, Val294, Trp286, Leu289, Tyr72	−4.6	3 *	Ala277, Gln119, Asn289, Asp70, Thr120, Ile69, Glu276
Neurotoxic substance												
Bisphenol A	−7.1	3	Asn331, Asn351, Asp134, Cys207, Ile131, Leu209, Leu350, Phe327, Ser110, Trp130, Trp355, Try358, Val215, Val331, Val354	−5.3	5	Arg37, His375, Lys35, Trp43, Trp377	−7.6	3	Arg296, Asp74, Leu76, Leu289, Phe295, Ser293, Thr75, Trp286, Tyr72, Tyr341, Val294	−6.4	4	Ala277, Asn68, Asn289, Asp70, Gln119, Glu276, Gly149, Pro285, Thr284
Neurotransmitters (Controls)												
Dopamine	−4.8	5 *	Ala113, Asp134, Cys207, Ile131, Trp130, Tyr358, Val208, Val354	−4.7	4 *	Arg220, Asp36, Asp224, Glu45, leu236, Thr44, Trp43	−5.5	3 *	Arg296, Gln291, Glu292, Leu289, Phe295, Ser293, Trp286, Tyr72, Tyr341, Val294	−4.7	5	Ala277, Asn68, Asn289, Asp70, Gln71, Gln119, Glu276, Gly149, Ile69, Thr120
Glutamic acid	−4.4	2	Asp134, Ile131, Leu209, Ser110, Trp130, Tyr358, Val208, Val354	−4.4	3 *	Asp224, Glu45, Ile38, Thr44, Tyr223	−5.0	3	Arg296, Leu289, Glu292, Tyr341, Gly342, Gln291, Phe297, Trp286, Phe295, Val294, Ser293,	−4.9	3	Asn68, Glu276, Asn83, Gln71, Asp70, Gln119, Ile69, Gly149, Thr120
n-Methyl-D-aspartic acid	−4.4	3	Asn351, Asp134, Cys207, Ile131, Ser110, Trp130, Tyr358, Val208, Val354	−4.5	2 *	Arg37, Arg220, Asp224, Glu45, leu38, Trp43, Tyr224	−4.8	3	Arg296, Val294, Gln291, Phe295, Leu289, Glu292, Ser293, Tyr341, Trp286	−4.7	3	Asn83, Ile69, Asp70, Asn68, Thr120, Gln67, Pro84, Gly116, Trp82, Gly121
Norepinephrine	−5.1	4	Ala113, Asp134, Ile131, Leu209, Ser110, Trp130, Tyr358, Val208, Val354	−4.8	4 *	Arg37, Arg220, Asp224, Glu45, Ile38, Thr44, Trp43	−6.0	3	Arg296, Leu289, Ser293, Gly342, Phe295, Tyr341, Trp286, Val294, Tyr72	−5.2	3	Thr120, Asn68, Ile69, Gln119, Asn289, Gly149, Asp70, Gln71, Ala277, Glu276
Serotonin	−5.6	3	Asp134, Cys207, Ile131, Leu209, Ser110, Trp130, Tyr358, Val135, Val208, Val354	−4.6	5	Ala34, Arg37, Asn237, Lys35	−6.2	3 *	Arg296, Ser293, Leu289, Val294, Tyr72, Phe295, Tyr341, Trp286	−5.2	3 *	Asp70, Gly149, Ala277, Phe290, Asn68, Thr120, Gln119, Ile69, Glu276, Asn289

BN = no. of formed bonds; BE = binding energy (Kcal/mol); * = subtract the unfavorable bonds from the detected number of formed bonds; AAs = amino acids; T4U = (1R)-8-chloro-1-methyl-2,3,4,5-tetrahydro-1H-3-benzazepine; LPD = L-dopamine; FAD = Flavin-adenine dinucleotide; NAG = 2-acetamido-2-deoxy-beta-D-glucopyranose; HUP = Huperzine A; MES = 2-(n-morpholino)-ethanesulfonic acid; GZ5 = (2~{R})-2-azanyl-~{N}-[6-[(6-chloranyl-1,2,3,4-tetrahydroacridin-9-yl)amino]hexyl]-3-(1~{H}-indol-3-yl)propanamide.

## Data Availability

The original contributions presented in this study are included in the article. Further inquiries can be directed to the corresponding author.

## Supplementary Materials

The following supporting information can be downloaded at: https://www.mdpi.com/article/10.3390/ph18040509/s1, Figure S1: Two- and three dimensional (2D and 3D) molecular interactions of: (A) Thymol; (B) α-Terpinene; and (C) p-Cymene identified in Thymus ciliatus with a neurotransmitter: control (D) Serotonin; and neurotoxic compound (E) Bisphenol A in the active site of human 5HT2C receptor (PDB ID: 8DPH), compared with T4U as a co-crystallized ligand (dimensions X: 15.9157, Y: 11.9977, Z: 14.1808); Figure S2: Two- and three dimensional (2D and 3D) molecular interactions of: (A) Thymol; (B) α-Terpinene; and (C) p-Cymene identified in Thymus ciliatus with neurotransmitters: controls (D) Dopamine; (E) Norepinephrine; and neurotoxic compound (F) Bisphenol A at the active site of monoamine oxidase (PDB ID: 8EEJ), compared with two co-crystalline ligands (FAD and LDP) (dimensions X: 28.4816, Y: 35.1878, Z: 22.8765); Figure S3: Two- and three dimensional (2D and 3D) molecular interactions of: (A) Thymol; (B) p-Cymene and (C) α– Terpinene identified in Thymus ciliatus with neurotransmitters: controls (D) Serotonin; (E) Norepinephrine; and neurotoxic compound (F) Bisphenol A at the active site of monoamine oxidase (PDB ID: 4EY5), compared with two co-crystallized ligands (NAG and HUP), (dimensions X: 21.8297, Y: 19.8175, Z: 19.5468; Figure S4: Two- and three dimensional (2D and 3D) molecular interactions of: (A) Thymol; (B) α-Terpinene; and (C) p-Cymene identified in *Thymus ciliatus* with neurotransmitters: control (D) Serotonin; (E) Norepinephrine and neurotoxic compound (F) Bisphenol A at the active site of monoamine oxidase (PDB ID: 6I0C), compared with three co-crystallized ligands (NAG, MES, GZ5), (dimensions X: 21.1196, Y: 18.7103, Z: 19.5559.
