# Supplementary material for: Role of Thymus ciliatus (Thyme) to Ameliorate the Acute Neurotoxicity Induced by Bisphenol A: In Vivo Supported with Virtual Study
Source: Pharmaceuticals (Basel). 2025 Mar 31;18(4):509. doi: 10.3390/ph18040509 (PMC12030012; doi:10.3390/ph18040509)
Supplement: Supplementary file 1 [file pharmaceuticals-18-00509-s001.zip › pharmaceuticals-3513201-supplementary.pdf]

Figures	Pages
**Figure S1.** Two- and three dimensional (2D and 3D) molecular interactions of: **(A)** Thymol; (**B)** α-Terpinene; and **(C)** p-Cymene identified in *Thymus ciliatus* with a neurotransmitter: control **(D)** Serotonin; and neurotoxic compound **(E)** Bisphenol A in the active site of human 5HT2C receptor (PDB ID: 8DPH), compared with T4U as a co-crystallized ligand (dimensions X: 15.9157, Y: 11.9977, Z: 14.1808).	2-4
**Figure S2.** Two- and three dimensional (2D and 3D) molecular interactions of: (**A**) Thymol; (**B**) α-Terpinene; and **(C)** p-Cymene identified in *Thymus ciliatus* with neurotransmitters: controls **(D)** Dopamine; **(E)** Norepinephrine; and neurotoxic compound **(F)** Bisphenol A at the active site of monoamine oxidase (PDB ID: 8EEJ), compared with two co-crystalline ligands (FAD and LDP) (dimensions X: 28.4816, Y: 35.1878, Z: 22.8765).	5-8
**Figure S3.** Two- and three dimensional (2D and 3D) molecular interactions of: (**A**) Thymol; **(B)** p-Cymene and **(C)** α– Terpinene identified in *Thymus ciliatus* with neurotransmitters: controls **(D)** Serotonin; **(E)** Norepinephrine; and neurotoxic compound **(F)** Bisphenol A at the active site of monoamine oxidase (PDB ID: 4EY5), compared with two co-crystallized ligands (NAG and HUP), (dimensions X: 21.8297, Y: 19.8175, Z: 19.5468.	9-12
**Figure S4.** Two- and three dimensional (2D and 3D) molecular interactions of: (**A**) Thymol; (**B**) α-Terpinene; and **(C)** p-Cymene identified in *Thymus ciliatus* with neurotransmitters: control **(D)** Serotonin; **(E)** Norepinephrine and neurotoxic compound **(F)** Bisphenol A at the active site of monoamine oxidase (PDB ID: 6I0C), compared with three co-crystallized ligands (NAG, MES, GZ5), (dimensions X: 21.1196, Y: 18.7103, Z: 19.5559.	13-17

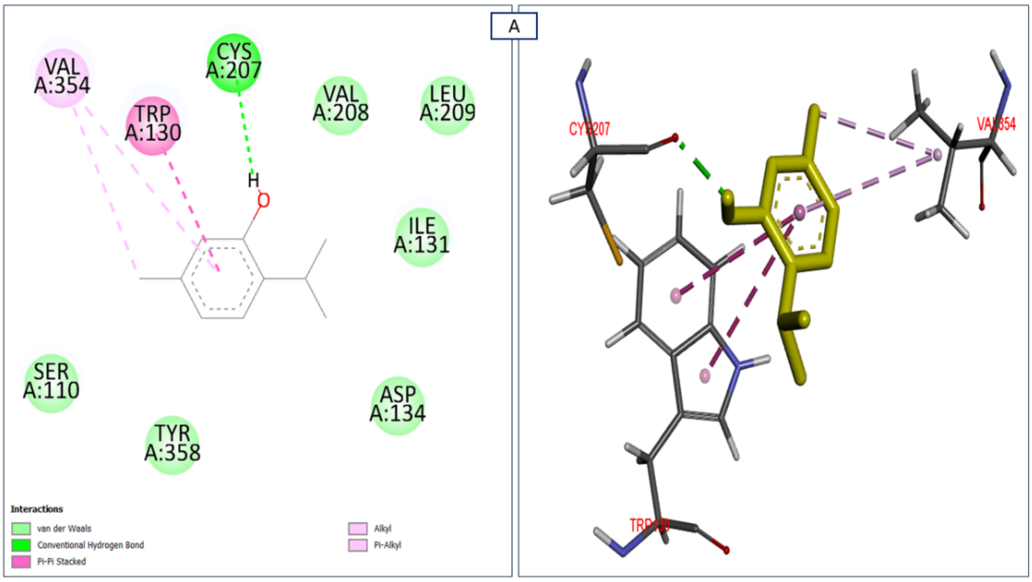
A
VAL A:354
CYS A:207
TRP A:130
VAL A:208
LEU A:209
ILE A:131
SER A:110
TYR A:358
ASP A:134
H
O
Interactions
van der Waals
Conventional Hydrogen Bond
Pi-Pi Stacked
Alkyl
Pi-Alkyl

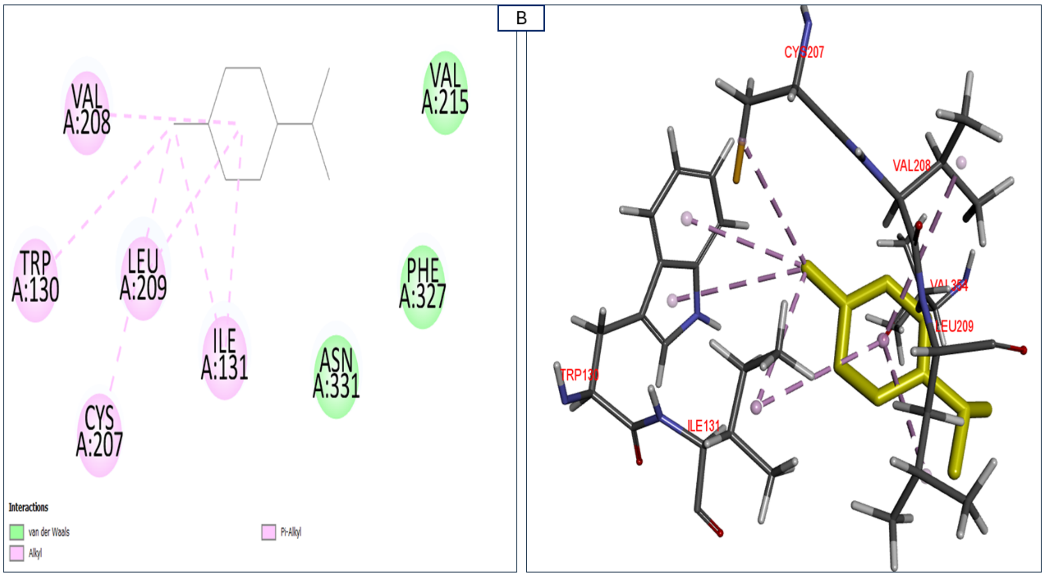
B
VAL A:208
VAL A:215
TRP A:130
LEU A:209
PHE A:327
ILE A:131
ASN A:331
CYS A:207
Interactions
van der Waals
Alkyl
Pi-Alkyl
CYS207
VAL208
LEU209
ILE131

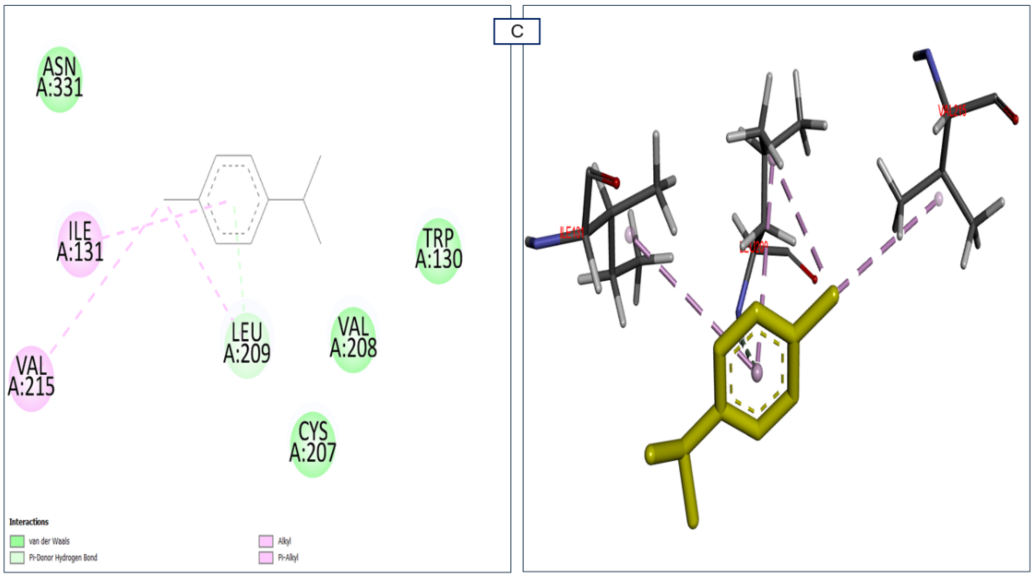
C
ASN A:331
ILE A:131
TRP A:130
LEU A:209
VAL A:208
VAL A:215
CYS A:207
Interactions
van der Waals
Pi-Donor Hydrogen Bond
Alkyl
Pi-Alkyl

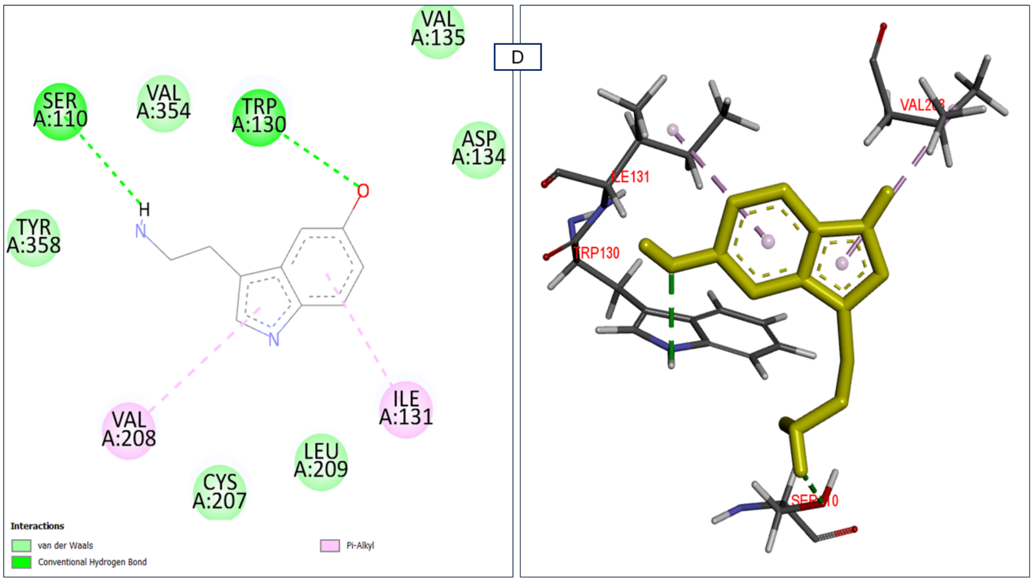
D
VAL A:135
SER A:110
VAL A:354
TRP A:130
ASP A:134
H
N
O
TYR A:358
N
ILE A:131
VAL A:208
LEU A:209
CYS A:207
Interactions
van der Waals
Conventional Hydrogen Bond
Pi-Alkyl
LE131
TRP130

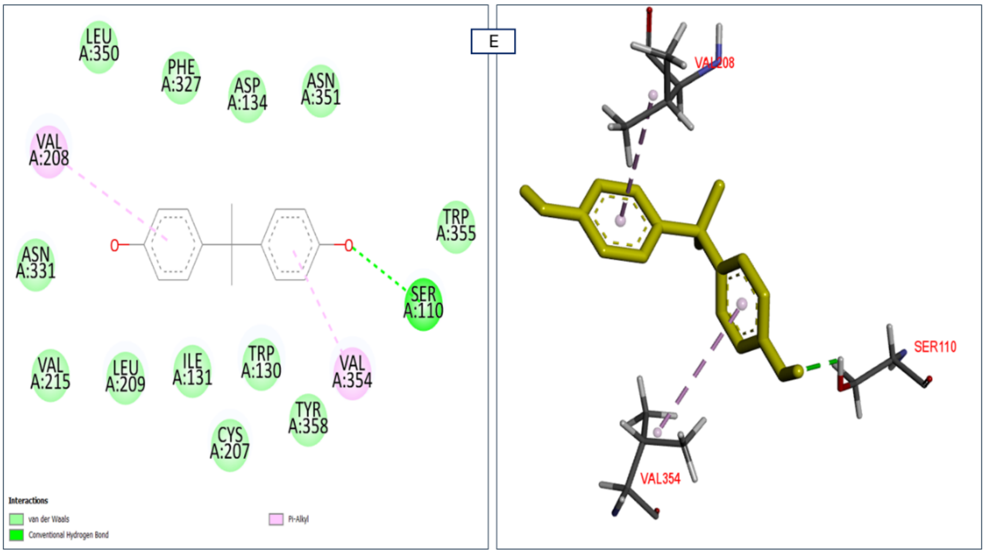

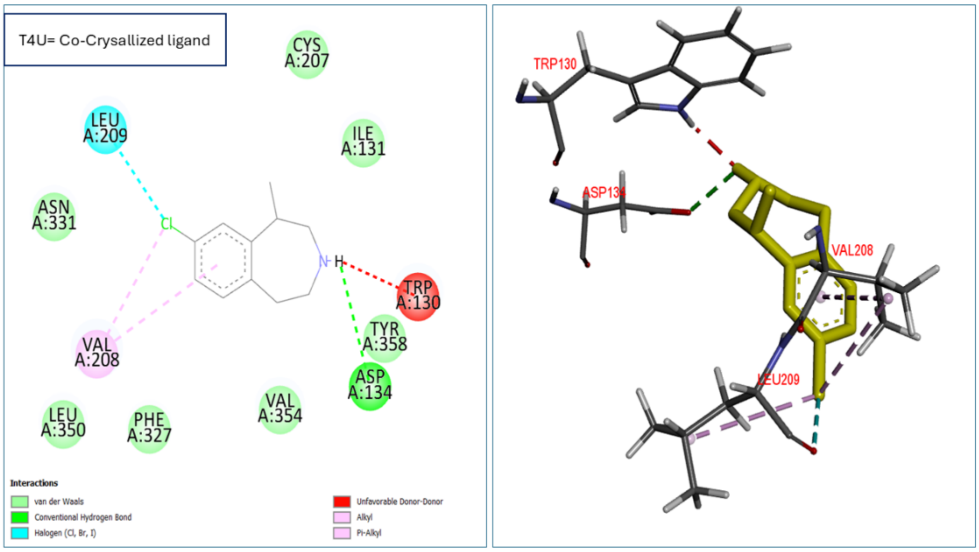

Figure S1. Two- and three dimensional (2D and 3D) molecular interactions of: (**A**) Thymol; (**B**) α-Terpinene; and (**C**) p-Cymene identified in *Thymus ciliatus* with a neurotransmitter: control (**D**) Serotonin; and neurotoxic compound (**E**) Bisphenol A in the active site of human 5HT2C receptor (PDB ID: 8DPH), compared with T4U as a co-crystallized ligand (dimensions X: 15.9157, Y: 11.9977, Z: 14.1808).

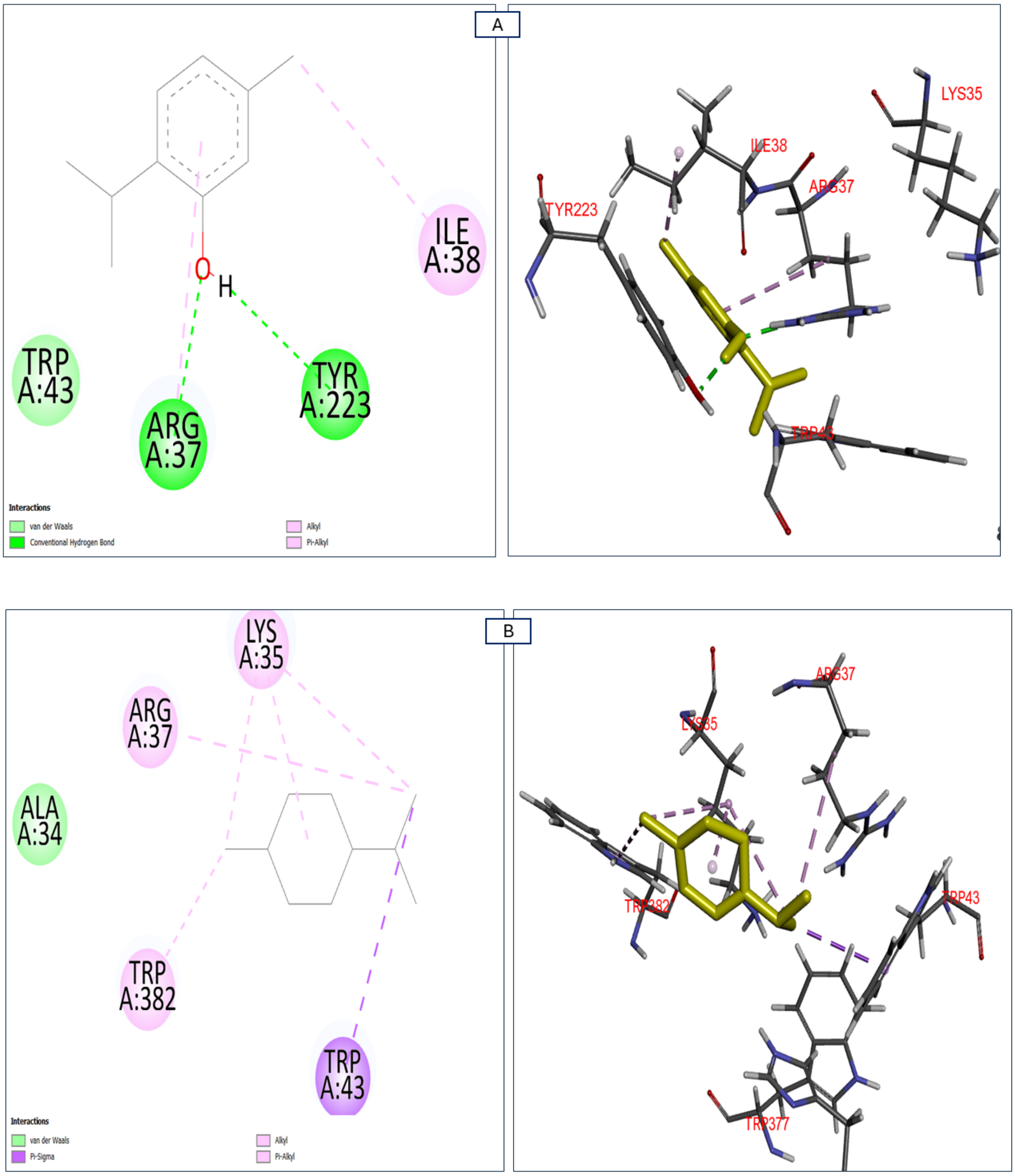
A
ILE A:38
TRP A:43
ARG A:37
TYR A:223
Interactions
van der Waals
Conventional Hydrogen Bond
Alkyl
Pi-Alkyl
LYS35
ILE38
ARG37
TYR223
TRP43
B
LYS A:35
ARG A:37
ALA A:34
TRP A:382
TRP A:43
Interactions
van der Waals
Pi-Sigma
Alkyl
Pi-Alkyl
ARG37
LYS35
TRP382
TRP43
TRP377

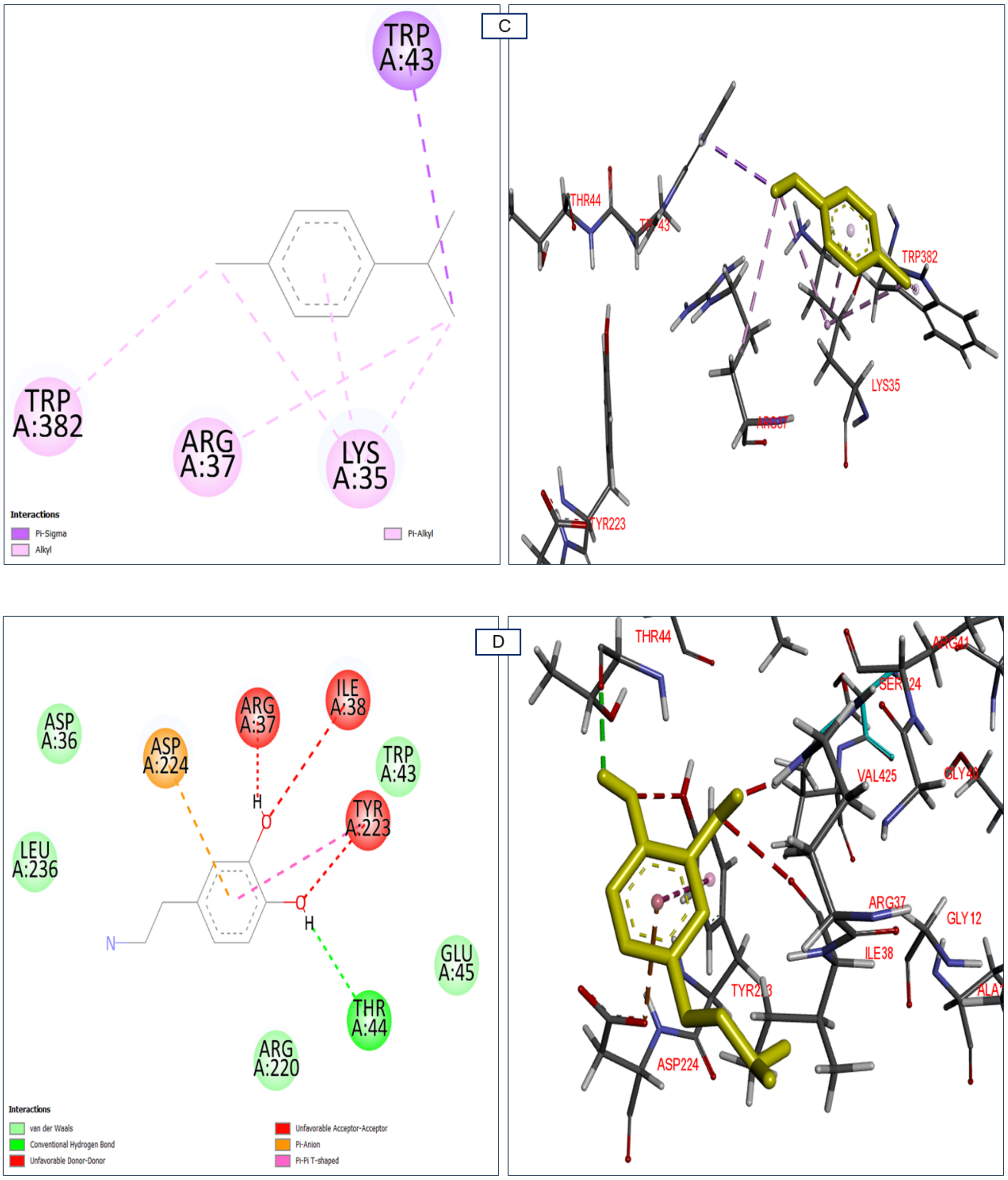
C
TRP A:43
TRP A:382
ARG A:37
LYS A:35
Interactions
Pi-Sigma
Alkyl
Pi-Alkyl
THR44
TRP382
LYS35
TYR223
D
ASP A:36
ASP A:224
ARG A:37
ILE A:38
TRP A:43
TYR A:223
LEU A:236
GLU A:45
THR A:44
ARG A:220
Interactions
van der Waals
Conventional Hydrogen Bond
Unfavorable Donor-Donor
Unfavorable Acceptor-Acceptor
Pi-Anion
Pi-Pi T-shaped
THR44
VAL425
ARG37
GLY12
ILE38
ASP224

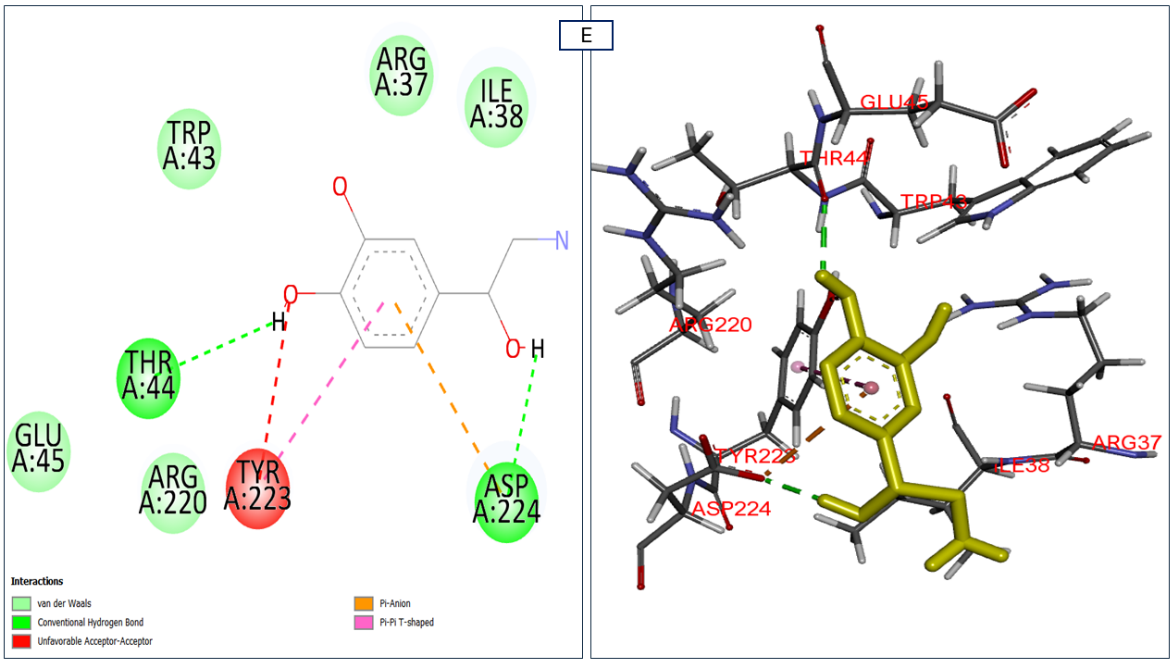
E
ARG A:37
ILE A:38
TRP A:43
THR A:44
GLU A:45
ARG A:220
TYR A:223
ASP A:224
Interactions
van der Waals
Conventional Hydrogen Bond
Unfavorable Acceptor-Acceptor
Pi-Anion
Pi-Pi T-shaped
GLU45
THR44
TRP43
ARG220
TYR223
ASP224
ILE38
ARG37

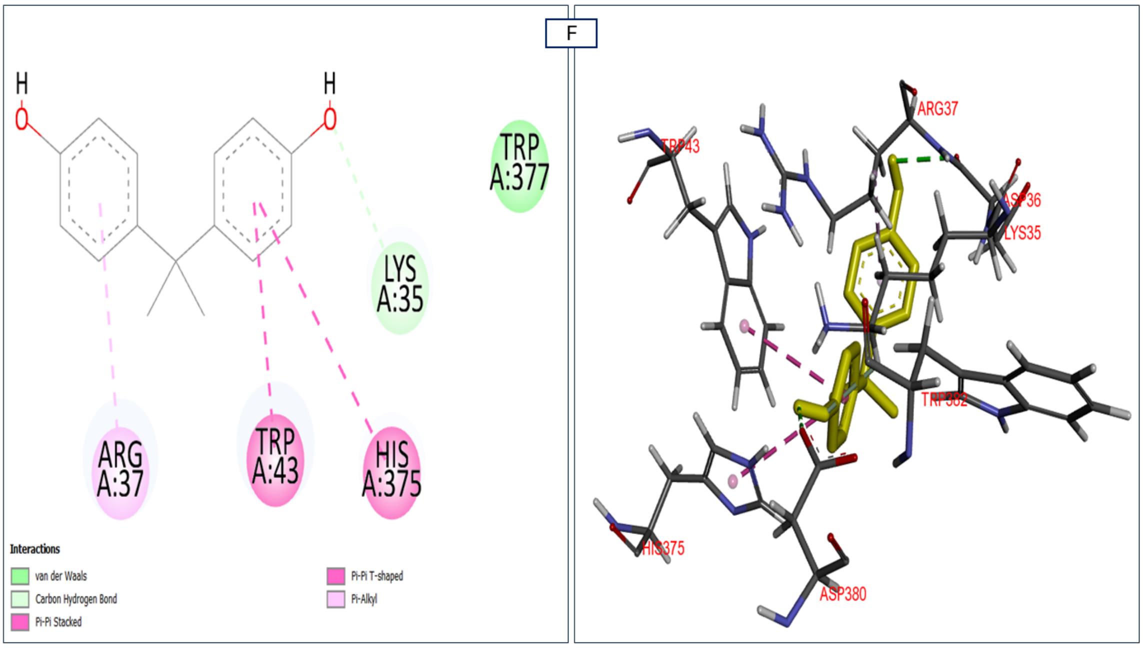
F
TRP A:377
LYS A:35
ARG A:37
TRP A:43
HIS A:375
Interactions
van der Waals
Carbon Hydrogen Bond
Pi-Pi Stacked
Pi-Pi T-shaped
Pi-Alkyl
ARG37
TRP43
LYS35
HIS375
ASP380

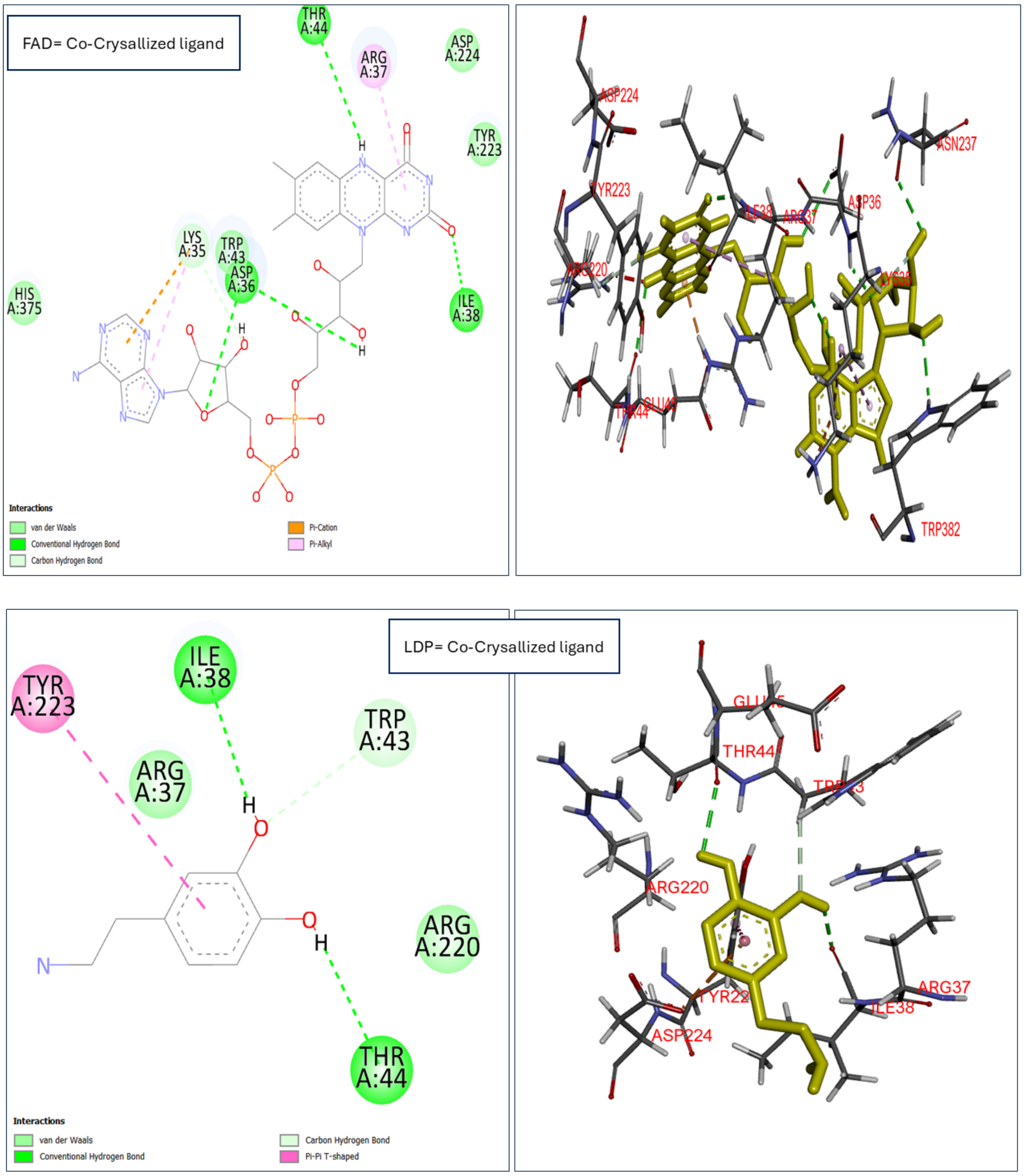

Figure S2. Two- and three dimensional (2D and 3D) molecular interactions of: (**A**) Thymol; (**B**) α-Terpinene; and (**C**) p-Cymene identified in *Thymus ciliatus* with neurotransmitters: controls (**D**) Dopamine; (**E**) Norepinephrine; and neurotoxic compound (**F**) Bisphenol A at the active site of monoamine oxidase (PDB ID: 8EEJ), compared with two co-crystalline ligands (FAD and LDP) (dimensions X: 28.4816, Y: 35.1878, Z: 22.8765).

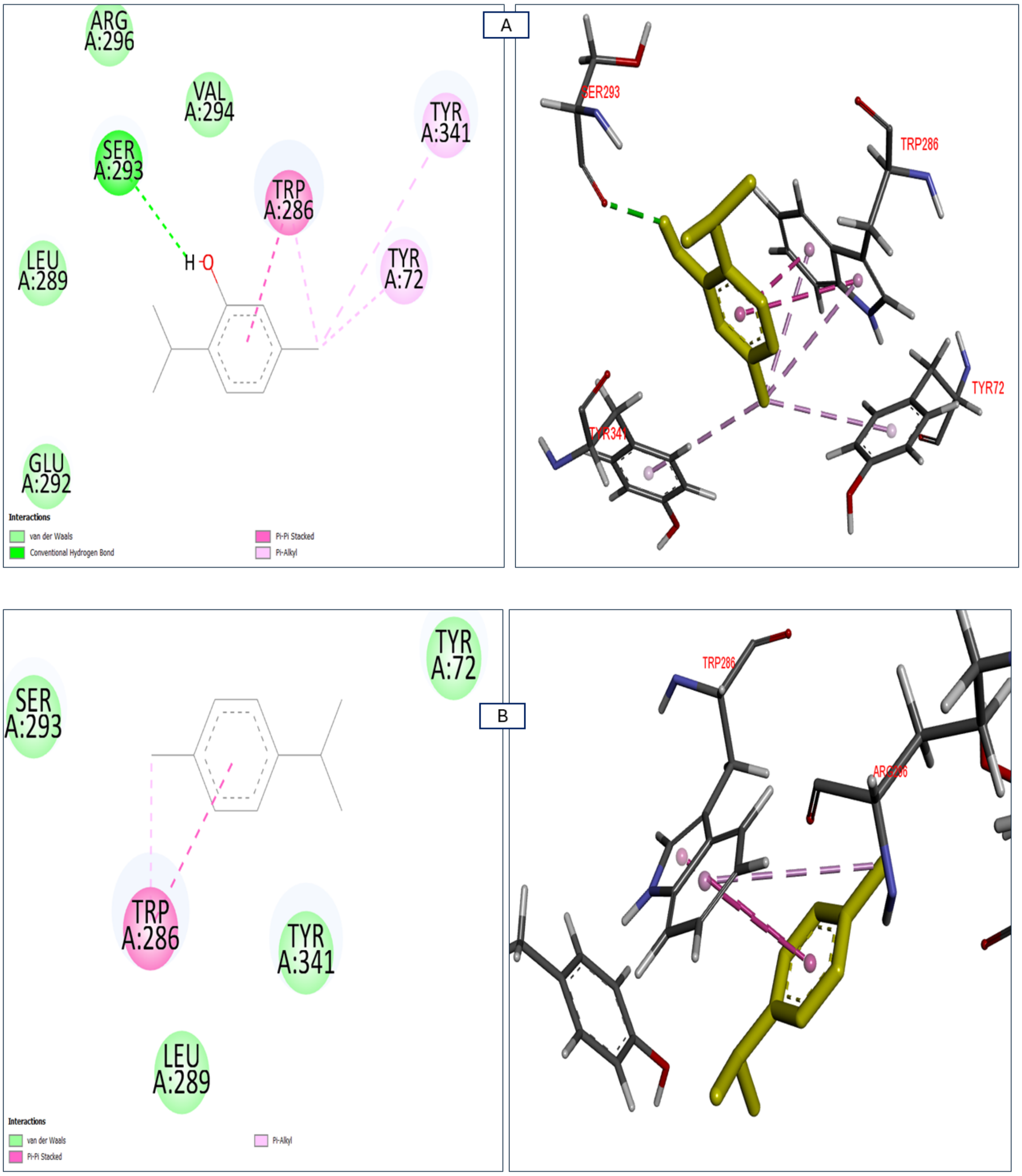
A
ARG A:296
VAL A:294
SER A:293
TRP A:286
TYR A:341
TYR A:72
LEU A:289
GLU A:292
H O
Interactions
van der Waals
Conventional Hydrogen Bond
Pi-Pi Stacked
Pi-Alkyl
SER293
TRP286
TYR72
TYR341
B
TYR A:72
SER A:293
TRP A:286
TYR A:341
LEU A:289
Interactions
van der Waals
Pi-Pi Stacked
Pi-Alkyl
TRP286
ARG296

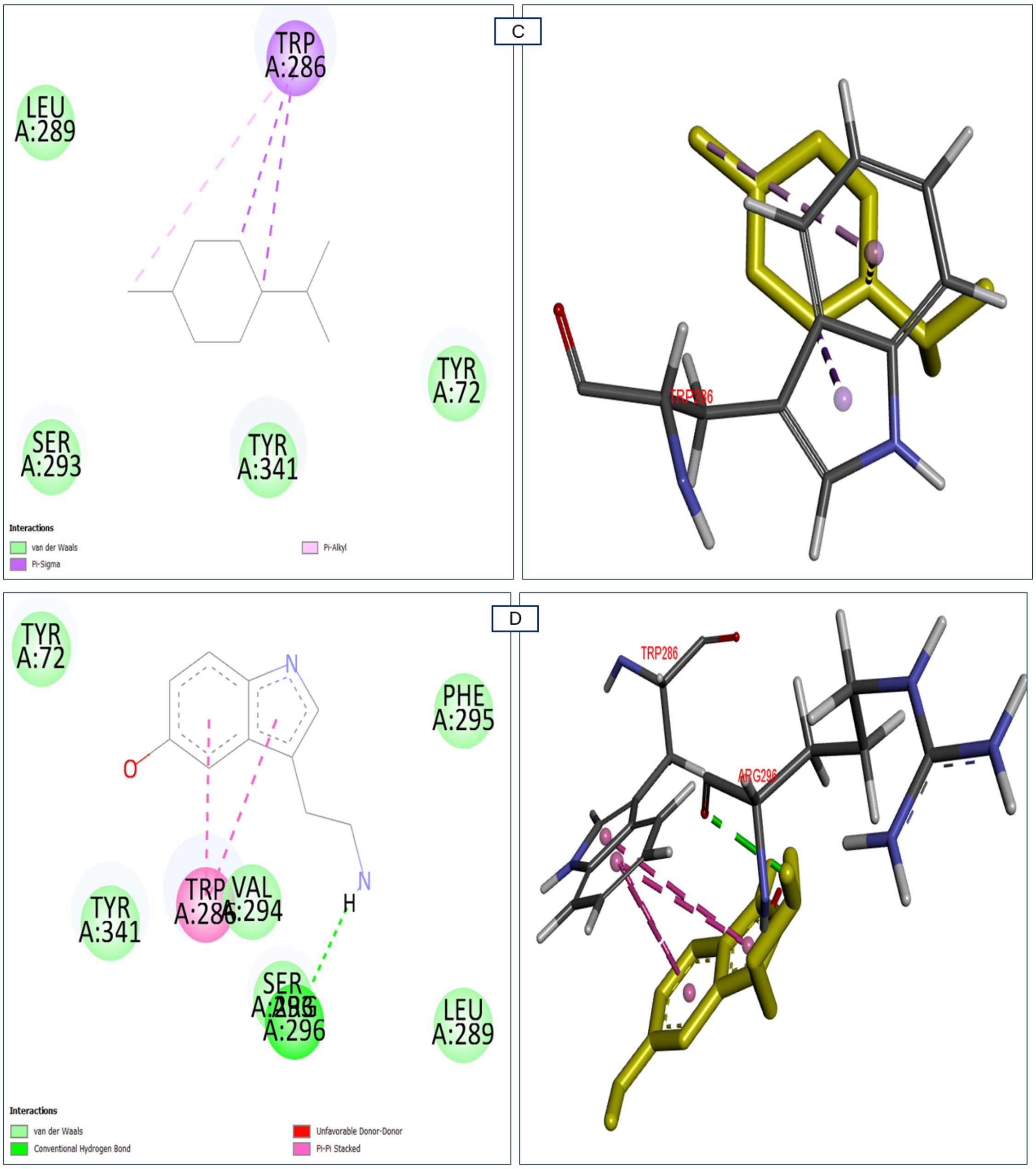
C
TRP A:286
LEU A:289
TYR A:72
SER A:293
TYR A:341
Interactions
van der Waals
Pi-Sigma
Pi-Alkyl
TRP286
D
TYR A:72
PHE A:295
O
N
H
TYR A:341
TRP A:286
VAL A:294
SER A:293
ARG A:296
LEU A:289
Interactions
van der Waals
Conventional Hydrogen Bond
Unfavorable Donor-Donor
Pi-Pi Stacked
TRP286
ARG296

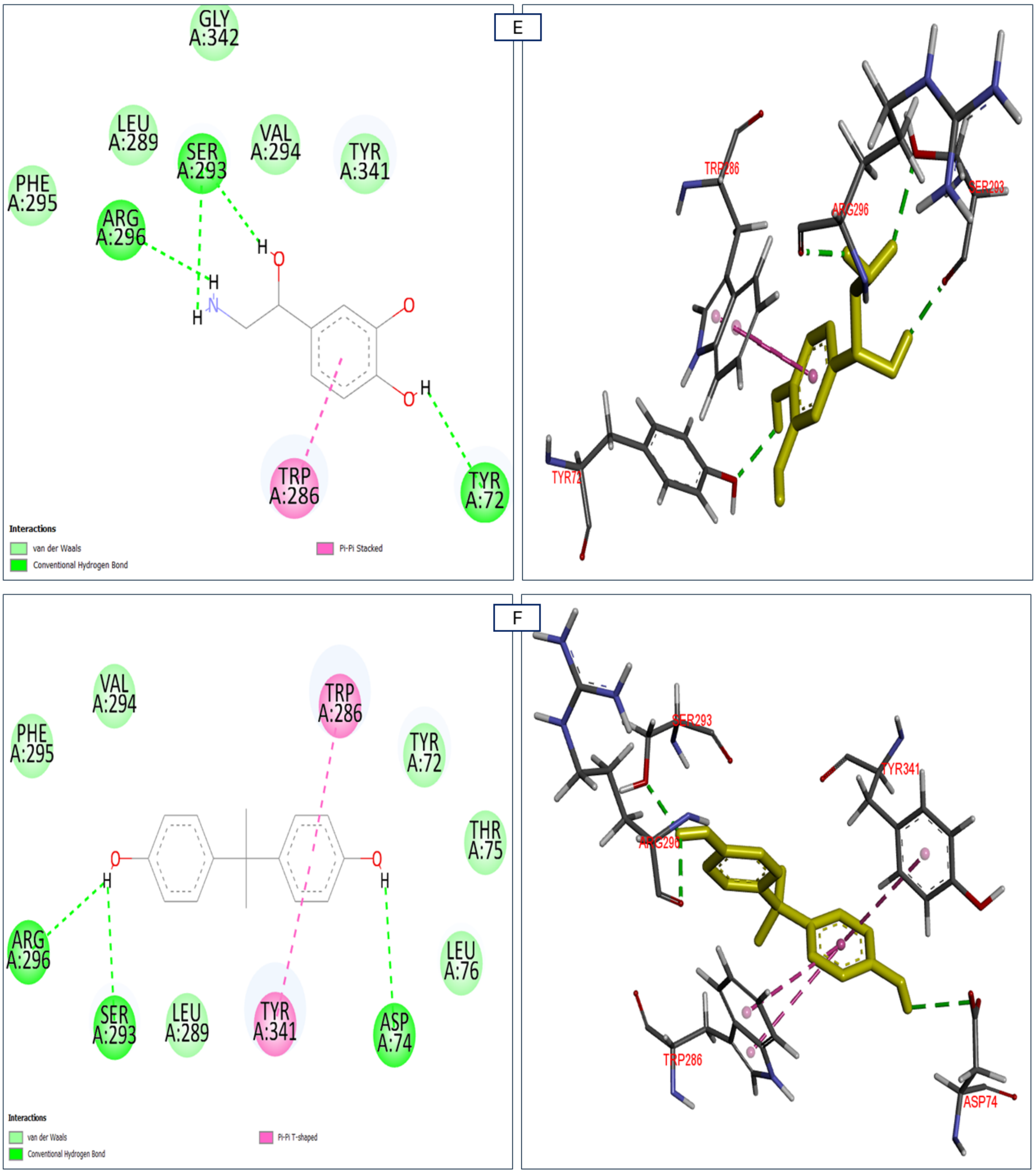
E
GLY A:342
LEU A:289
SER A:293
VAL A:294
TYR A:341
PHE A:295
ARG A:296
TRP A:286
TYR A:72
Interactions
van der Waals
Conventional Hydrogen Bond
Pi-Pi Stacked
TRP286
ARG296
SER293
TYR72
F
VAL A:294
PHE A:295
TRP A:286
TYR A:72
THR A:75
ARG A:296
SER A:293
LEU A:289
TYR A:341
ASP A:74
LEU A:76
Interactions
van der Waals
Conventional Hydrogen Bond
Pi-Pi T-shaped
SER293
TYR341
TRP286
ASP74

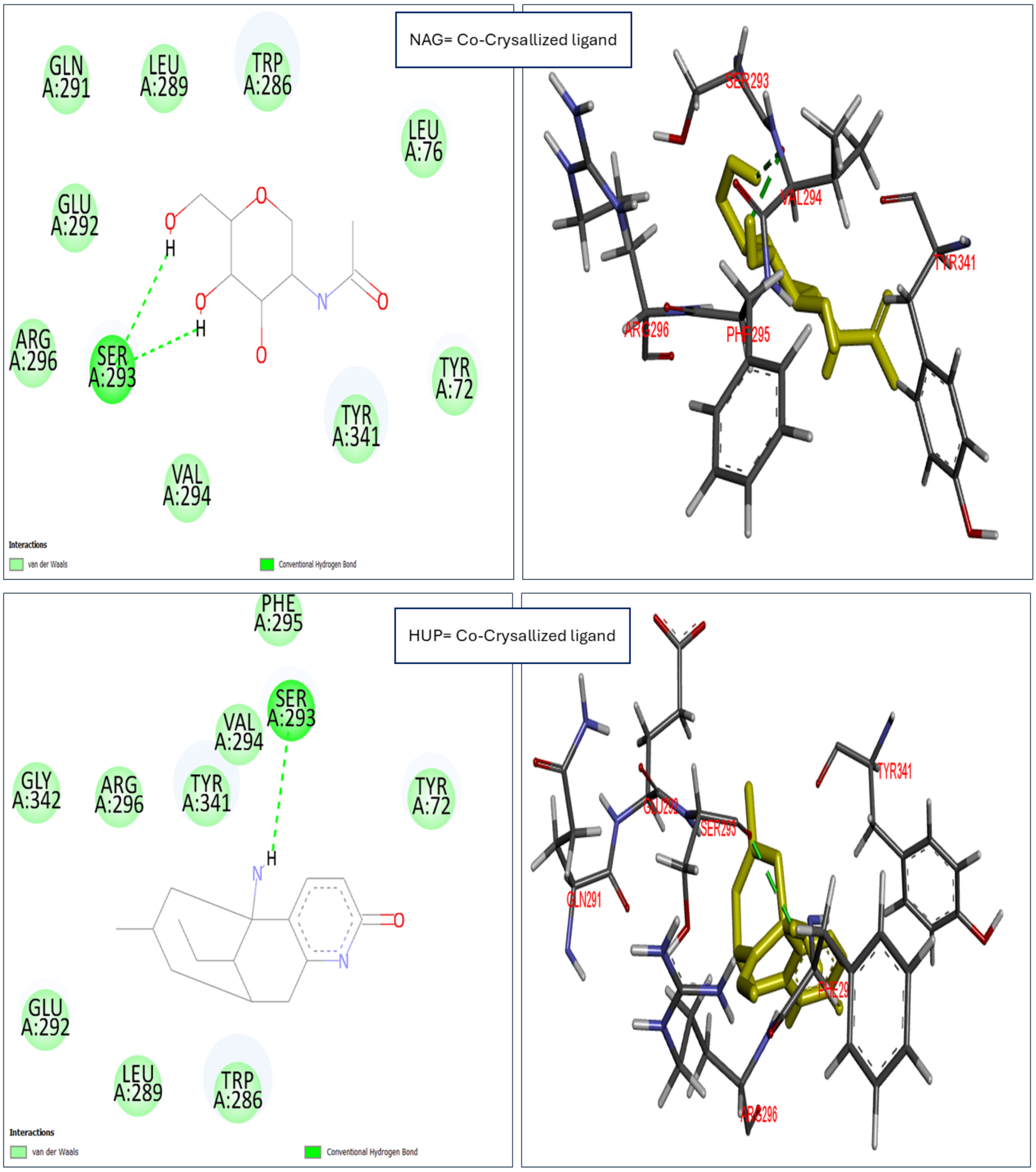

Figure S3. Two- and three dimensional (2D and 3D) molecular interactions of: (**A**) Thymol; (**B)** p-Cymene and (**C**) α– Terpinene identified in *Thymus ciliatus* with neurotransmitters: controls (**D**) Serotonin; (**E**) Norepinephrine; and neurotoxic compound (**F**) Bisphenol A at the active site of monoamine oxidase (PDB ID: 4EY5), compared with two co-crystallized ligands (NAG and HUP), (dimensions X: 21.8297, Y: 19.8175, Z: 19.5468.

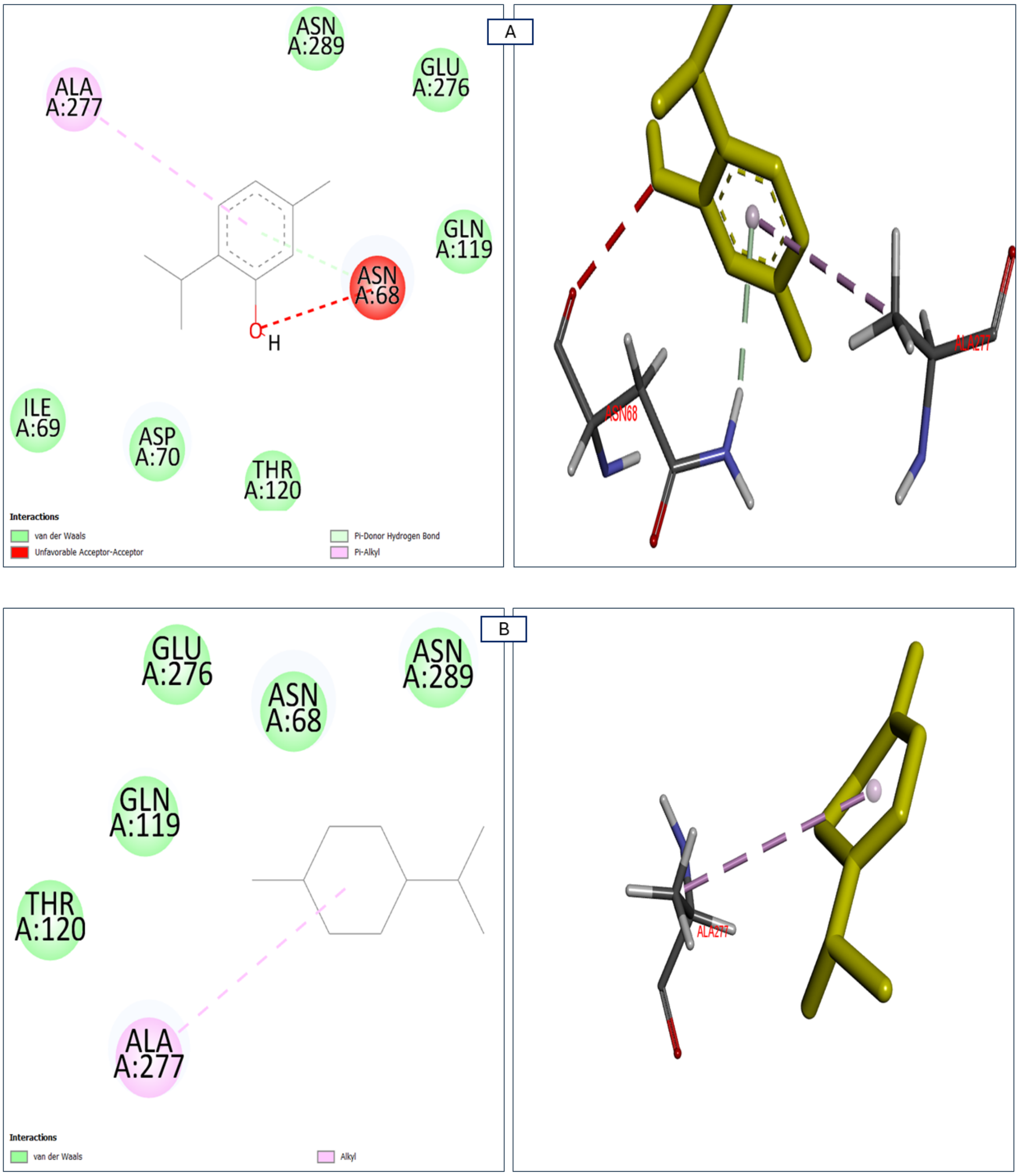
A
ASN A:289
GLU A:276
ALA A:277
GLN A:119
ASN A:68
O
H
ILE A:69
ASP A:70
THR A:120
Interactions
van der Waals
Unfavorable Acceptor-Acceptor
Pi-Donor Hydrogen Bond
Pi-Alkyl
ASN68
ALA277
B
GLU A:276
ASN A:68
ASN A:289
GLN A:119
THR A:120
ALA A:277
Interactions
van der Waals
Alkyl
ALA277

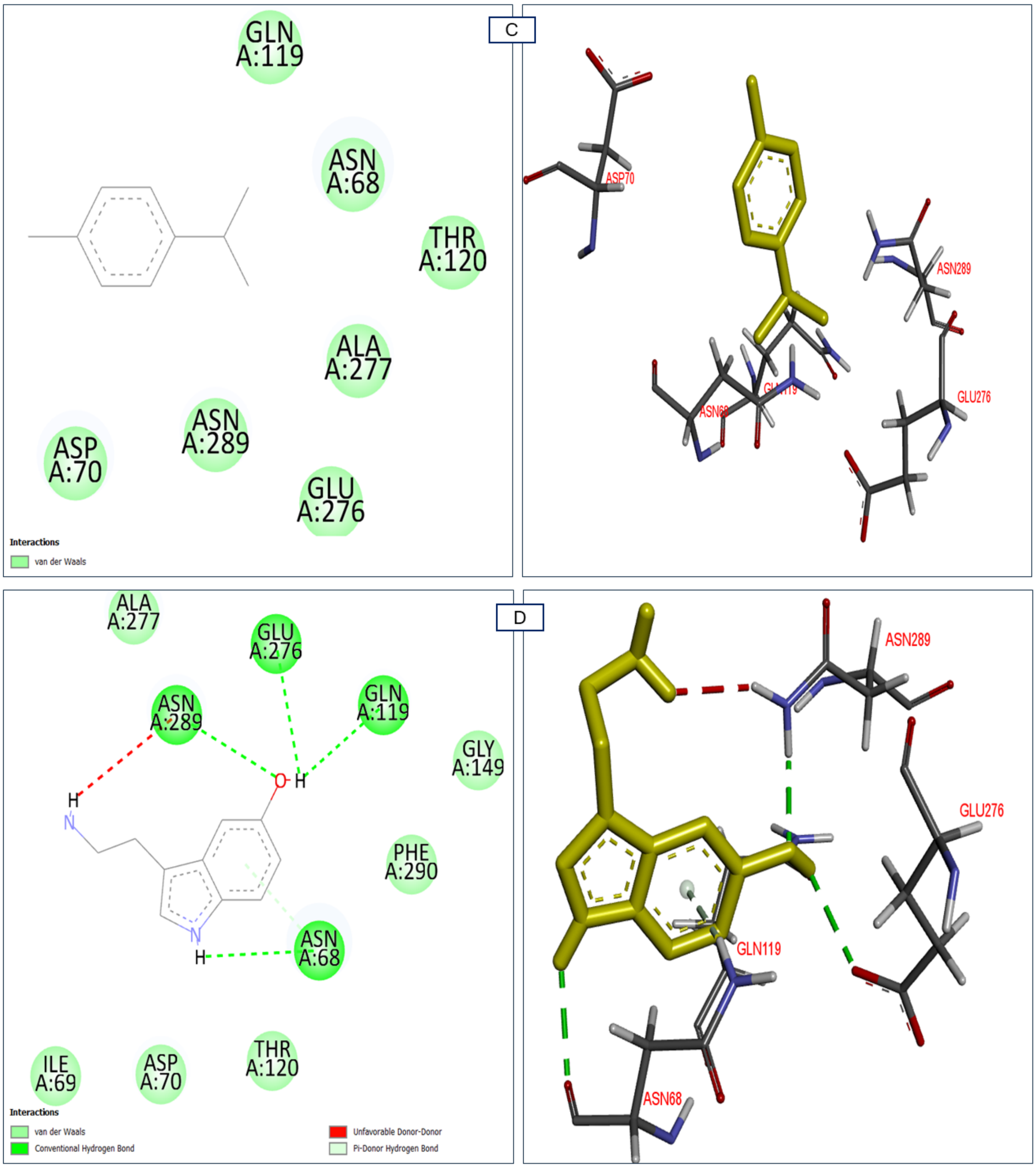
C
GLN A:119
ASN A:68
THR A:120
ALA A:277
ASN A:289
ASP A:70
GLU A:276
Interactions
van der Waals
ASP70
ASN289
GLU276
D
ALA A:277
GLU A:276
ASN A:289
GLN A:119
GLY A:149
PHE A:290
ASN A:68
THR A:120
ILE A:69
ASP A:70
Interactions
van der Waals
Conventional Hydrogen Bond
Unfavorable Donor-Donor
Pi-Donor Hydrogen Bond
ASN289
GLU276
GLN119
ASN68

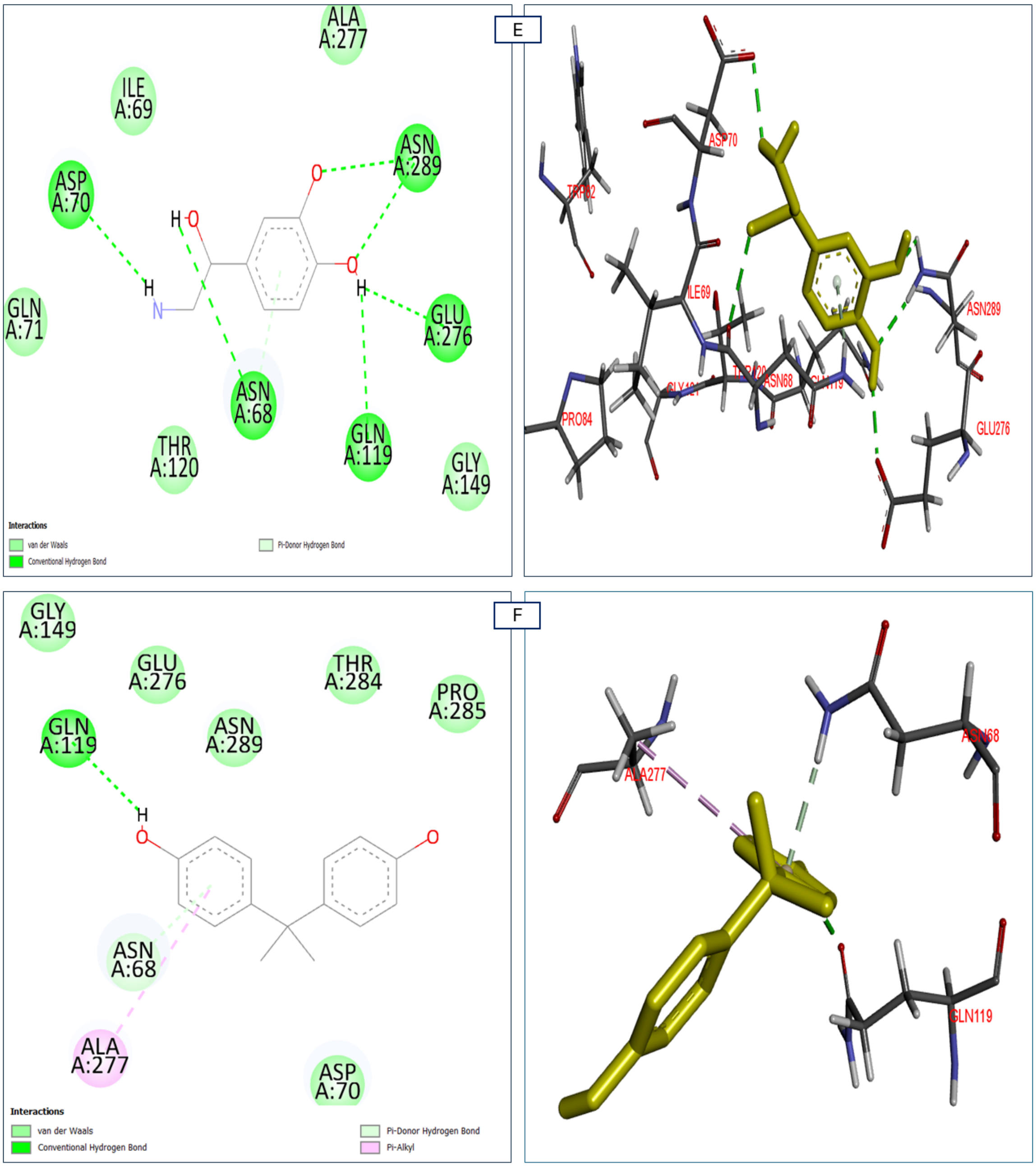
E
ALA A:277
ILE A:69
ASP A:70
ASN A:289
GLN A:71
GLU A:276
ASN A:68
GLN A:119
THR A:120
GLY A:149
Interactions
van der Waals
Conventional Hydrogen Bond
Pi-Donor Hydrogen Bond
ASP70
TRP82
ILE69
ASN289
ASN68
PRO84
GLU276
F
GLY A:149
GLU A:276
THR A:284
PRO A:285
GLN A:119
ASN A:289
ASN A:68
ALA A:277
ASP A:70
Interactions
van der Waals
Conventional Hydrogen Bond
Pi-Donor Hydrogen Bond
Pi-Alkyl
ALA277
ASN68
GLN119

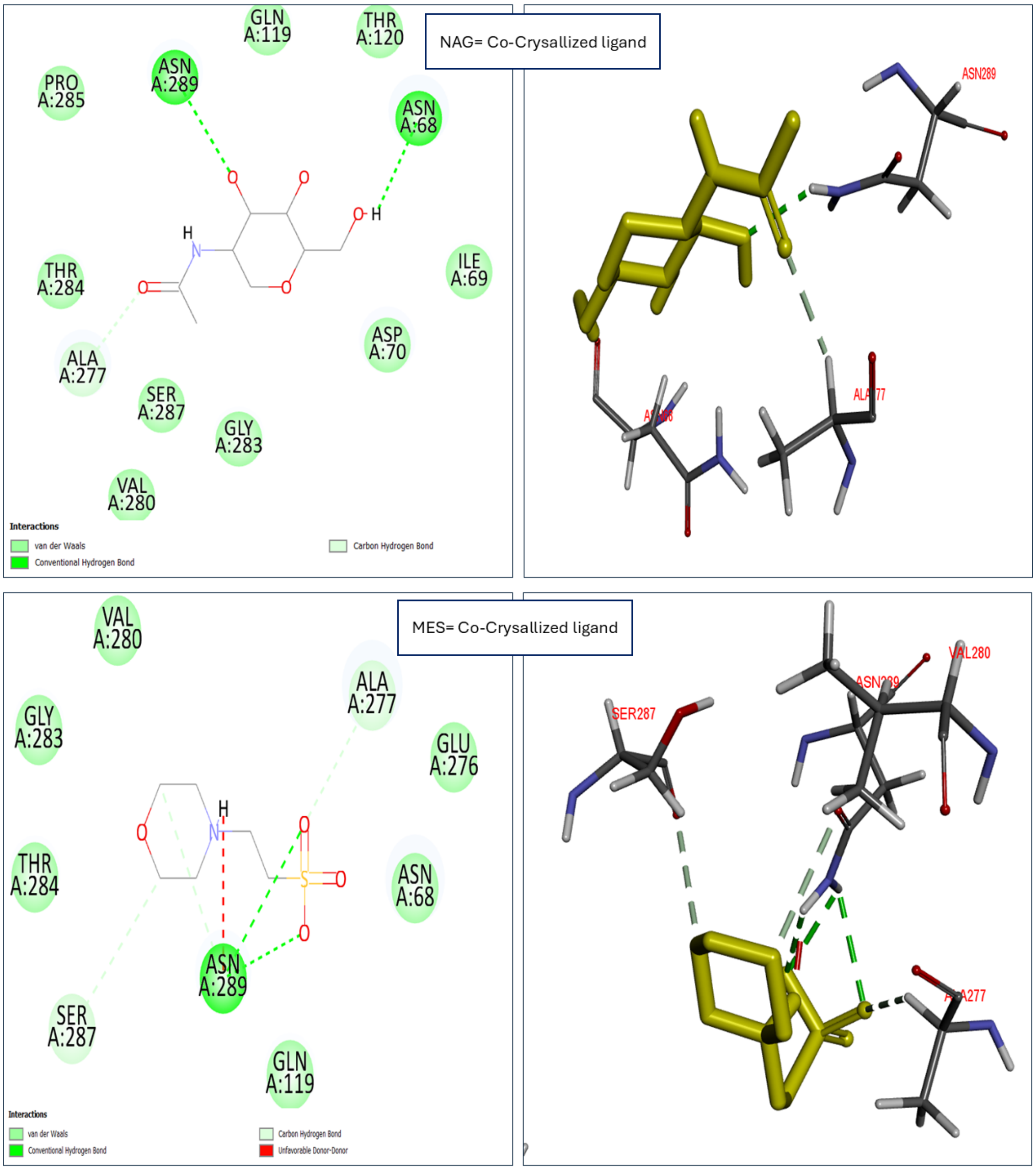
NAG= Co-Crysallized ligand
GLN A:119
THR A:120
PRO A:285
ASN A:289
ASN A:68
THR A:284
ILE A:69
ALA A:277
ASP A:70
SER A:287
GLY A:283
VAL A:280
Interactions
van der Waals
Conventional Hydrogen Bond
Carbon Hydrogen Bond
ASN289
MES= Co-Crysallized ligand
VAL A:280
GLY A:283
ALA A:277
GLU A:276
THR A:284
ASN A:68
ASN A:289
SER A:287
GLN A:119
Interactions
van der Waals
Conventional Hydrogen Bond
Carbon Hydrogen Bond
Unfavorable Donor-Donor
SER287
VAL280

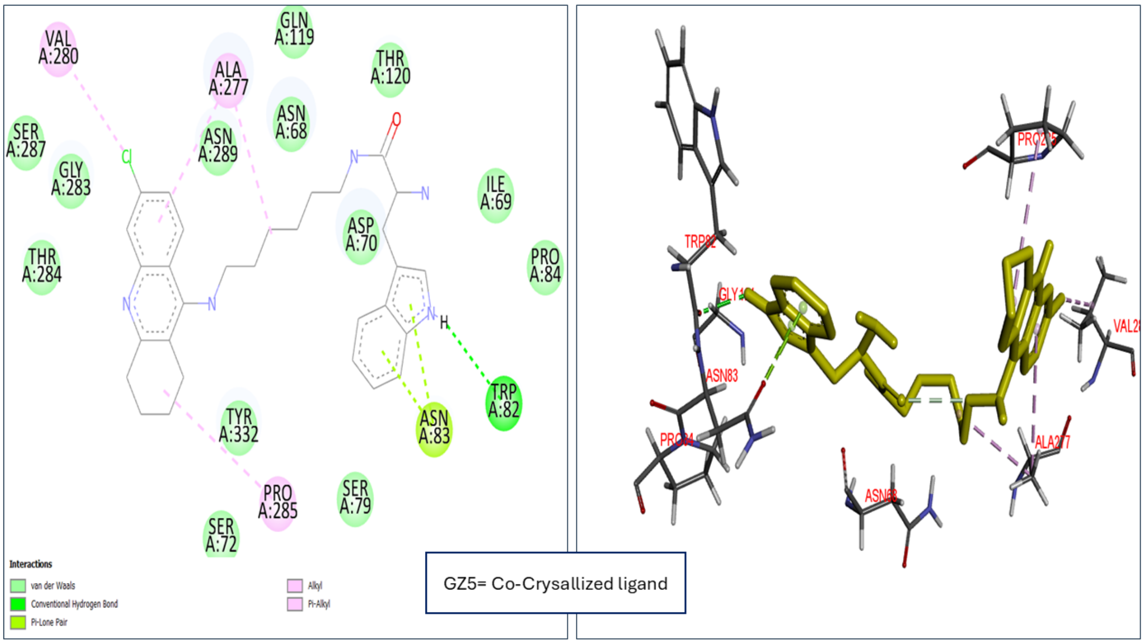

Figure S4. Two- and three dimensional (2D and 3D) molecular interactions of: (**A**) Thymol; (**B**) α-Terpinene; and (**C**) p-Cymene identified in *Thymus ciliatus* with neurotransmitters: control (**D**) Serotonin; (**E**) Norepinephrine and neurotoxic compound (**F**) Bisphenol A at the active site of monoamine oxidase (PDB ID: 6I0C), compared with three co-crystallized ligands (NAG, MES, GZ5), (dimensions X: 21.1196, Y: 18.7103, Z: 19.5559.
